# Selective filtering of relevant sensory signals in parietal cortex

**DOI:** 10.21203/rs.3.rs-8564427/v1

**Published:** 2026-04-01

**Authors:** Constanza D. Bassi, Saloni Saxena, Mabry Smyer, James Ryan, Chengcheng Huang, Caroline A. Runyan

**Affiliations:** 1 Department of Neuroscience, University of Pittsburgh, Pittsburgh, PA, USA.; 2 Center for the Neural Basis of Cognition, University of Pittsburgh, Pittsburgh, PA, USA.; 3 Department of Mathematics, University of Pittsburgh, Pittsburgh, PA, USA.

**Keywords:** cortical circuits, perceptual decision-making, population coding

## Abstract

Animals must flexibly adjust their behavior to respond appropriately in changing behavioral situations, in order to survive and thrive. The posterior parietal cortex (PPC) has been implicated in flexible decision-making, as it encodes sensory stimuli depending on their behavioral relevance [1] and is required to adapt to changes in stimulus-response contingencies in behavioral tasks [2]. Here, we show that while mice performed an auditory decision-making task, the temporal organization of neural activity differed across excitatory and inhibitory interneuron subtypes, with pyramidal neurons sparsely encoding task-related information. Sensory responses of all cell types were modulated according to behavioral relevance in the task, and signatures of this modulation were evident even before the presentation of sensory stimuli. Population activity patterns preceding stimulus presentation predicted the strength of sensory responses and even the mouse’s behavioral accuracy in the task. A network model revealed that randomly organized inhibitory connectivity could replicate the selective filtering of sensory responses, but that context-dependent inputs to the network must be targeted to neurons responding to behaviorally relevant cues, and avoid those responding to irrelevant cues. Our results reveal a selective filtering mechanism in cortical circuits underlying flexible decision-making.

## Introduction

Perceptual decision making requires the brain to integrate sensory evidence to inform the appropriate behavioral response for the current situation. Posterior parietal cortex is a key hub in the brain’s decision-making network, where neural activity reflects ongoing evidence accumulation [3–6]. One proposed mechanism includes competing neural ensembles that integrate evidence toward different choice outputs [3], which is supported by the patterns of noise correlations among excitatory and inhibitory neurons [7], and the mutual, disynaptic inhibition between excitatory neurons encoding opposing choices[8]. In the natural environment, a given sensory stimulus may be relevant for behavior in one context but not another, requiring dynamic gating of sensory integration for decision making.

Neural responses in PPC to sensory stimuli depend on their behavioral relevance in both primates [1, 9] and rodents [2, 10], and on the behavioral state of the animal [11]. Thus sensory inputs in PPC appear to be modulated or gated according to their relevance, before being accumulated. This gating could arise from circuit mechanisms that bias activity of neurons according to the relevance of the stimuli they encode. This could be implemented through top-down or neuromodulatory inputs, which strongly impact local inhibitory interneurons [12–17].

## Results

Inhibitory interneurons are well positioned to implement a flexible, state dependent gate for incoming sensory signals. Inhibitory cell types such as parvalbumin-expressing (PV) and somatostatin-expressing (SOM) interneurons differentially regulate the flow of excitation in cortical circuits through their different connectivity motifs. PV neurons provide fast perisomatic inhibition to excitatory neurons with similar inputs [18, 19], shaping spike timing and response gain [20–22], whereas SOM neurons modulate dendritic integration regulating synaptic integration and response reliability [20, 23, 24]. Furthermore, the responses of PV, SOM, and other inhibitory interneurons are highly sensitive to shifts in neuromodulatory tone [13, 16, 17], enabling interneurons to mediate state-dependent regulation of sensory responses [15].

Here, we examined the effects of behavioral relevance on the encoding of sensory stimuli in PV, SOM, and putative pyramidal neurons, using two-photon calcium imaging of these cell types simultaneously, behavioral state manipulations, and network modeling. We hypothesized that behavioral state would engage local inhibitory circuits, with different impacts on PV and SOM interneurons, to modulate the processing of incoming sensory signals in PPC. We show that selective sensory filtering is implemented in PPC through both the enhancement of task-relevant sensory ensembles and suppression of irrelevant ensembles even before sensory stimuli are presented. A network model required selective contextual drive to the neurons encoding the relevant sensory input to recapitulate this computation. Surprisingly, the model did not require structured, opponent local inhibition between neurons encoding relevant and irrelevant sensory cues. Our results provide a circuit mechanism for flexible sensory gating in parietal cortex.

### Cell-type-specific representations of task variables in an auditory decision-making task

**Figure 1.**
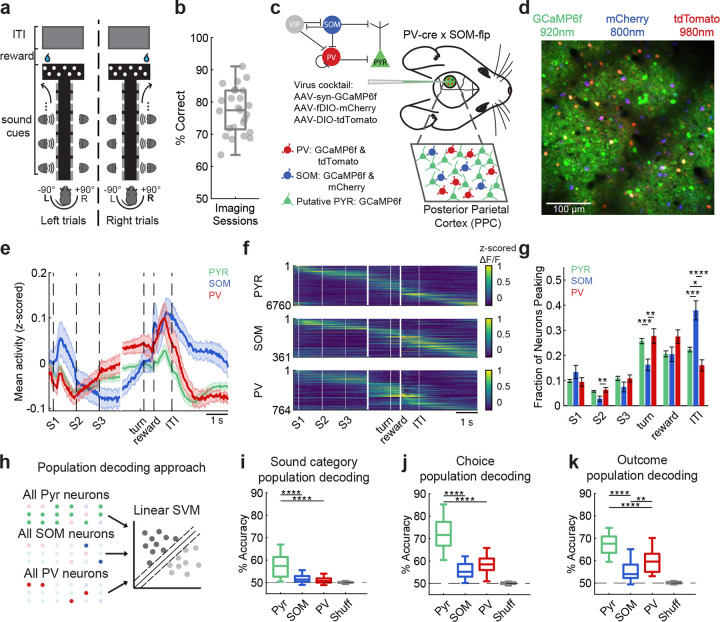
Cell-type-specific encoding of task variables in an auditory decision-making task (a) Task schematic. (b) Task performance during imaging sessions (gray dots correspond to individual sessions; *n* = 25 datasets from 6 mice, mean ± s.e.m. = 77.4±1.4%). (c) Labeling strategy for simultaneous imaging of PV, SOM, and Pyr neurons. See Methods Section 0.7 (d) Example field of view, showing separability of tdTomato and mCherry to identify SOM and PV neurons. Composite image was made by combining images collected at different excitation wavelengths. Green: green channel at 920 nm excitation. Blue: red channel at 800 nm excitation. Red: red channel at 980 nm excitation. mCherry+ neurons appear more blue while tdTomato neurons appear more red. All neurons are green as they express GCaMP. (e) Trial averaged activity of each cell type (shading indicates s.e.m. across imaging sessions; *n* = 25). (f) Trial-average of z-scored activity of all recorded PPC neurons (Pyr *n* = 6760, SOM *n* = 361, PV *n* = 764), sorted by time of peak activity. Neurons were sorted using one half of the trials and displayed using the other half. (g) Fraction of neurons with peak activity during each trial epoch, separated by cell type (mean ± s.e.m. across datasets; paired permutation test *n* = 25, Extended Table 1). “S1”, “S2”, and “S3” includes sound onset to offset for each repeat; “turn”includes 1 s before turn onset to reward onset; “reward” spans reward onset to the start of the intertrial interval; “ITI” covers the remaining post-trial period. (h) Population decoding approach. Activity from all Pyr, all SOM, or all PV neurons used to train linear SVMs to decode sound category, choice, or trial outcome. See Methods Section 0.12. (i) Sound category decoding accuracy for population decoders trained on Pyr, SOM, or PV neurons (mean classification accuracy in the 450 ms following event onset; *n* = 23 datasets). (j) Choice decoding accuracy (same as i; *n* = 23 datasets). (k) Outcome decoding accuracy (same as i; *n* = 19 datasets). In all panels, errorbars and shading indicate s.e.m. ****: *p* < 0.0001, ***: *p* < 0.001, **: *p* < 0.01, *: *p* < 0.05. Exact *p* values are provided in Extended Table 1.

We trained mice (SOM-Flp^*het*^xPV-Cre^*het*^) on a head-fixed, navigation-based auditory localization task in virtual reality (VR) [25] that requires PPC activity [26]. While mice navigated the VR T-maze, a sound was presented from either the left (−90°) or right (+90°) speaker, signaling the arm of the maze that would be rewarded on that trial (Figure 1a). Mice performed the task above chance levels (77.4% ±1.4% correct, mean ± s.e.m.; Figure 1b). In each mouse’s posterior parietal cortex (PPC), we expressed the green calcium indicator GCaMP6f in all neurons and the red fluorophores tdTomato in PV neurons and mCherry in SOM neurons [27] (Figure 1c, see Methods Section 0.7, Extended Data Figure 1). We used two-photon calcium imaging to characterize the spike-related activity of PV, SOM, and putative pyramidal (Pyr) neurons in layer 2/3 (Figure 1c–d; *n* = 25 datasets from 6 mice, collected at 150–250 *μ*m depth). A typical field of view contained 270 ± 14 Pyr, 31 ± 2 PV, and 14 ± 1 SOM neurons.

Trial-averaged activity revealed distinct temporal dynamics across cell types. While the first sound cue presentation evoked a transient population response in all cell types, mean Pyr and PV activity then ramped through the middle of the trial before becoming relatively suppressed during the inter-trial interval (ITI). In contrast, mean SOM activity was higher during the first stimulus presentation (S1), suppressed in the middle of the trial, and then increased and remained higher than other cell types through the ITI (Figure 1e). Considering the activity of single neurons, activity in all neuron types was transient, but collectively tiled the full trial, consistent with previous reports of PPC population activity during navigation-based decision tasks [5, 25, 28, 29] (Figure 1f). Although each cell type included neurons that were active in every trial epoch (sound cue presentation, turn, reward, and ITI), the fraction of neurons with peak responses in specific epochs differed (Figure 1g). A smaller proportion of SOM neurons had peak activity during the choice period (defined as the time window spanning 1 second before the mouse turned into one of the maze arms (‘turn’) to the onset of the reward period (‘reward’)), while a higher proportion of SOM neurons had peak activity during the ITI compared to other cell types (Figure 1g and Extended Table 1; ‘S2’ SOM–PV *p* = 0.0076; ‘turn’ Pyr–SOM *p* = 0.0004, SOM–PV *p* = 0.0011; ‘ITI’ Pyr–SOM *p* = 0.0003, SOM–PV *p* = 0.0002; paired permutation test). These distinct activity dynamics suggest functional specializations for PV and SOM neurons in shaping the population code in PPC.

**Extended Data Figure 2:**
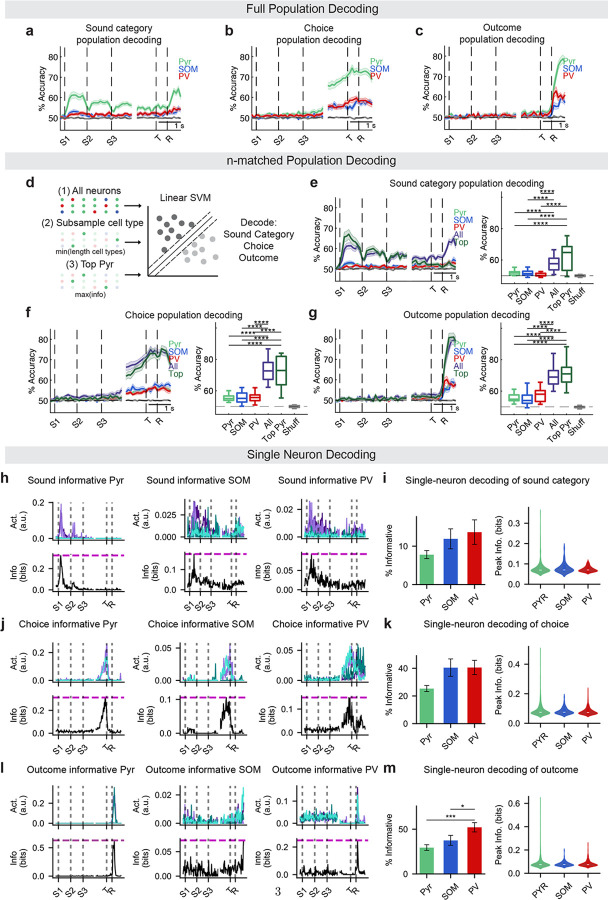
**, related to** Figure 1. Full and *n*-matched population decoders and single cell decoders. (a) Sound category classification accuracy of SVM decoders trained on all Pyr, SOM, or PV populations (*n* = 23). (b) Choice classification accuracy of SVM decoders trained on all Pyr, SOM, or PV populations (*n* = 23). (c) Outcome classification accuracy of SVM decoders trained on all Pyr, SOM, or PV populations (*n* = 19). (d) Neurons were grouped in three ways: (1) all recorded neurons (“All”), (2) randomly subsampled neurons matched to the lowest cell-type count (“Pyr,” “SOM,” “PV”), and (3) top-ranked Pyr neurons with peak information that also matched the lowest cell-type count (“Top Pyr”). Linear SVMs were trained on 90 ms non-overlapping bins to decode sound category, choice, or outcome. (e) *Left:* Classification accuracy of SVM decoders trained on all neurons (purple), subsampled Pyr (light green), subsampled SOM (blue), subsampled PV (red), or top Pyr (dark green). *Right:* Mean classification accuracy in the 450 ms after event onset (*n* = 23 datasets). (f) Same as (b), but for choice decoding (*n* = 23 datasets). (g) Same as (b), but for outcome decoding (*n* = 19 datasets). (h) Example sound-informative neurons per cell type. *Top*: Averaged deconvolved activity across trial types (dark green: left sound, left choice, correct; teal: left sound, right choice, incorrect; dark purple: right sound, left choice, incorrect; light purple: right sound, right choice, correct). *Bottom*: Instantaneous information about sound category (magenta line indicates peak information), from Bayesian decoder based on single neuron’s activity. (i) Sound decoding: Left: Percent of informative neurons across datasets (mean ± SEM, *n* = 23 datasets). Right: Peak information content across cell types (PYR = 474/6209, SOM = 38/330, PV = 98/724 neurons). (j) Same as (h) but for choice decoding. (k) Choice decoding: Same as (i), but for choice information (PYR = 1482/6209, SOM = 124/330, PV = 290/724 neurons; *n* = 23 datasets). (l) Same as (h) but for outcome decoding. (m) Outcome decoding: Same as (i), but for outcome information (PYR = 1423/5090, SOM = 114/294, PV = 306/604 neurons; *n* = 19 datasets). For all panels, n.s. indicates no significance; *, *p* < 0.05; **, *p* < 0.01; ***, *p* < 0.001; ****, *p* < 0.0001. Exact *p*-values are provided in Extended Table 1.

To quantify the maximum task-related information in each cell type’s activity, we trained and tested population decoders (cross-validated linear support vector machines) to predict sound location, choice direction, and trial outcome from the activity of each cell type’s full population (Figure 1h; see Methods Section 0.12). Population activity from all three cell types carried significant information about all three task variables (sound category: *p*_pyr–shuff_ = 1.64 × 10^−8^, *p*_som–shuff_ = 3.42 × 10^−4^, *p*_pv–shuff_ = 0.0211; choice: *p*_pyr–shuff_ = 6.64–10–9, *p*_som–shuff_ = 1.75–10–8, *p*_pv–shuff_ = 7.56–10–9; outcome: *p*_pyr–shuff_ = 1.48 × 10^−7^, *p*_som–shuff_ = 3.45 × 10^−6^, *p*_pv–shuff_ = 1.48 × 10^−7^; unpaired permutation test; Figure 1ik). However, population decoders based on Pyr neurons consistently outperformed PV or SOM decoders (Figure 1i; sound category: Pyr–SOM *p* = 0.0002, SOM–PV *p* = 0.0646, Pyr–PV *p* = 0.0001; Figure 1j; choice: Pyr–SOM *p* = 0.0001, SOM–PV *p* = 0.0160, Pyr–PV *p* = 0.0001; Figure 1k; outcome: Pyr–SOM *p* = 0.0001, SOM–PV *p* = 0.0073, Pyr–PV *p* = 0.0002; Extended Data Figure 2a–c).

The higher decoding accuracy in Pyr populations could result from a dense coding scheme where many neurons encode small amounts of information, or from a sparse coding regime where a small number of neurons are highly informative. To distinguish between these possibilities, we further analyzed information coding at the levels of populations and single neurons. First, we built population decoders using matching numbers of PV, SOM and Pyr neurons (which required randomly subsampling Pyr and PV neurons to match the smaller *n* of SOM neurons; *n*_sound category_ = 13.96±5.11, *n*_choice_ = 13.96±5.11, *n*_outcome_ = 15 ± 4.86, mean ± s.d.). These *n*-matched population decoders performed comparably when using PV, SOM, or Pyr neurons (sound category: Pyr–SOM *p* = 0.9102, Pyr–PV *p* = 0.0048, SOM–PV *p* = 0.0187; choice: Pyr–SOM *p* = 0.9572, Pyr–PV *p* = 0.9362, SOM–PV *p* = 0.9085; outcome: Pyr–SOM *p* = 0.7637, Pyr–PV *p* = 0.1010, SOM–PV *p* = 0.0998; paired permutation test; Extended Data Figure 2d–g), consistent with previous studies [7]. However, when Pyr neurons were sampled using the *n* most informative neurons instead of randomly, the resulting decoders performed similarly to those trained on the full Pyr population (Top Pyr–All: sound category *p* = 0.0406, choice *p* = 0.6093, outcome *p* = 0.5761; paired permutation test; Extended Data Figure 2d–g).

Next, we built Bayesian decoders based instead on each single neuron’s activity (see Methods Section 0.13, Extended Data Figure 2h–m). Surprisingly, information content related to stimulus and choice was similar in individual Pyr, PV, and SOM neurons (see Extended Data Figure 2i,k). Especially in Pyr neurons, the distribution of information across single neurons was heavy tailed, indicating that a small fraction of of Pyr neurons carried disproportionately high task-related information.

Together, these results suggest that although task-relevant variables are encoded across all cell types in PPC, population-level decoding is dominated by a limited subset of highly informative Pyr neurons. These highly informative Pyr neurons are not often sampled in the random, n-matched population decoders (Extended Data Figure 2d–g), but are likely responsible for the higher Pyr population decoding performance when using all neurons for decoding.

### Selective filtering of auditory signals in active vs passive listening

Sensory responses in PPC neurons are strongly modulated by task engagement [10] and by behavioral relevance [1]. We therefore asked how behavioral context modulates auditory responses in PPC, and how this modulation is reflected in local circuit activity. We hypothesized that PV and SOM neurons, which provide feedforward and lateral inhibition to excitatory neurons and also directly interact (Figure 1c), could provide a local circuit mechanism for a long-range, task-specific input to modulate the encoding of stimulus information in PPC.

**Extended Data Figure 3:**
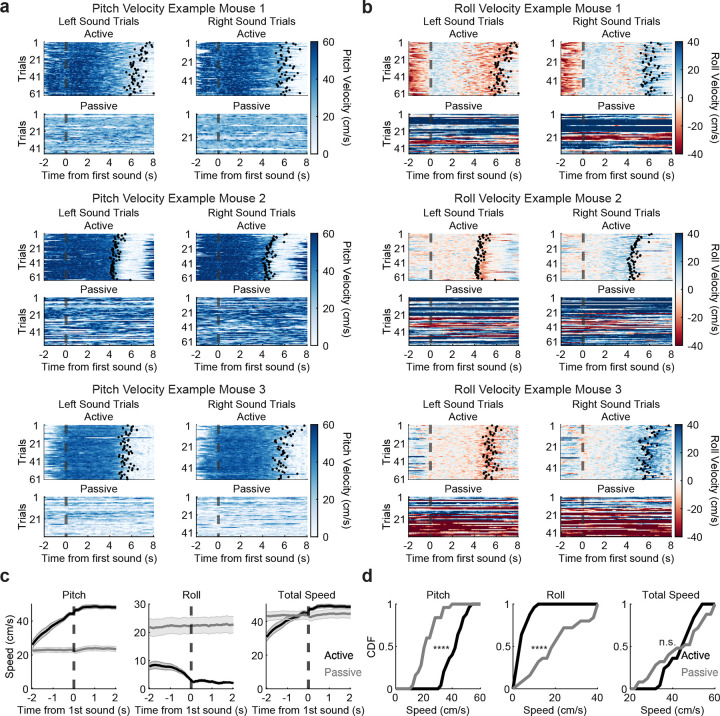
**, related to** Figure 2 Mice had different running patterns in active and passive contexts but similar overall speed. (a) Example running velocity changes in the pitch axis, which drove forward progression in VR in the active context, in three example mice in active (top) and passive (bottom) trials. Dotted line indicates timing of first sound onset. Black dots indicate timing of turn onset during each trial in the active context. Only correct trials are plotted in active listening context. (b) Same as (a) but for running velocity in the roll axis, which was used for turning left/right in VR Tmaze arms in the active listening context. Negative roll = turning left; positive roll = turning right. (c) Average speed (absolute value of velocity) across contexts aligned to sound onset across pitch, roll, total speed (combination of pitch + roll; mean ± s.e.m. across datasets, *n* = 25). (d) CDF of speed in each axis averaged along the 4 seconds centered around sound onset across contexts (*n* = 25 datasets, paired permutation test: *p*_pitch_ = 9.9999 × 10^−5^, *p*_roll_ = 9.9999 × 10^−5^, *p*_both_ = 0.8758).

**Figure 2.**
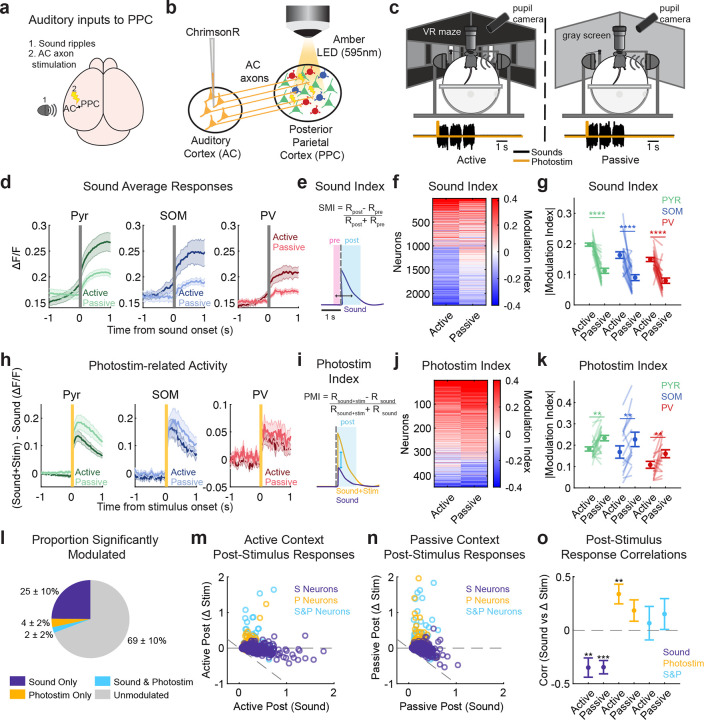
Auditory-driven responses in PPC were modulated by behavioral context. (a) Two auditory signals were delivered to PPC neurons: natural sound stimulation delivered via the ears and direct photostimulation of auditory (AC) axons terminating in PPC. (b) ChrimsonR was expressed in auditory cortex (AC), and AC axons were photostimulated in PPC with a 595 nm LED. (c) Auditory stimuli were delivered in two behavioral contexts: active listening in the task of Figure 1, and passive listening. Black traces: timing of sound stimuli. Yellow traces: timing of photostimulations, which occurred only at the time of the first sound repeat, in 33% of trials. (d) Trial-averaged responses of sound-responsive neurons with positive sound-evoked responses, in active (dark) and passive (light) contexts, for each cell type (shading indicates s.e.m. across datasets; Pyr *n* = 25, SOM *n* = 16, PV *n* = 15. Only datasets with at least 1 significant neuron were included). (e) Definition of sound modulation index. (f) Heatmap of the modulation indices for significantly sound-responsive neurons in active or passive contexts (*n* = 2248 Pyr, SOM, and PV neurons). Neurons were sorted by mean modulation index across contexts. (g) Mean absolute value of the sound modulation index for each cell type (Pyr *n* = 25, SOM *n* = 23, PV *n* = 24 datasets. Only datasets with at least 1 significant neuron were included). Lines link values from the same imaging session. (h) Difference between trial-averaged responses during stim+sound and sound-only trials, shown only for photostim-responsive neurons with a positive difference (shading indicates s.e.m. across datasets; Pyr *n* = 24, SOM *n* = 11, PV *n* = 12. Only datasets with at least 1 significant neuron were included). (i) Definition of photostimulation modulation index. (j) Same as (f), but showing each neuron’s photostimulation modulation index in both contexts (*n* = 446 neurons), including only neurons with significant responses to photostimuli in the spontaneous context (See Methods Section 0.10). (k) Mean absolute value of the photostimulation modulation index for each cell type ( Pyr *n* = 24, SOM *n* = 14, PV *n* = 16 datasets. Only datasets with at least 1 significant neuron were included). (l) Proportion of significantly modulated neurons classified as Sound only, Photostim only, and Sound& Photostim (mean ± s.d. across datasets; n = 24 datasets). (m-n) Neuron-level post-stimulus responses to sound-only vs. photostimulation-related activity, quantified as Δ Stim = (sound + stim) sound, shown separately for active (m) and passive (n) contexts. Neurons are grouped by functional class: Sound (S) = 1992, Photostim (P) = 261, Sound and Photostim (S & P) = 185 neurons. (o) Correlation between sound-evoked and photostimulation-evoked post-stimulus responses for each neuron class, plotted as the mean ± s.e.m. across datasets (S=24, P=23, S & P=19 datasets. Only datasets with at least 1 significant neuron were included). Asterisks indicate correlations significantly different from zero. For all panels, n.s. means no significance; *, *p* < 0.05; **, *p* < 0.01; ***, *p* < 0.001; ****, *p* < 0.0001. Error bars indicate s.e.m. across datasets.

We manipulated the relevance of auditory cues in two complementary ways. First, in addition to the sound stimuli used in the task, we introduced a task irrelevant ‘auditory’ input to PPC, which was a photostimulation of ChrimsonR-expressing axons from auditory cortex (AC) innervating PPC (Figure 2a–b, Methods Section 0.6). In 33% of trials, a 100ms AC axon photostimulation was delivered simultaneously with the first sound cue (Figure 2c, black and orange lines). Second, we presented the sound cues and AC axon photostimulations in two behavioral contexts: *active listening* (the auditory localization task of Figure 1), in which the sounds but not photostimuli were used to inform behavioral choices, and *passive listening*, in which the same stimuli were presented but the mouse was not performing any behavioral task (Figure 2c). In the passive context, mice did not exhibit task-related behavior, such as preferential turning toward the side that played the auditory cue (Extended Data Figure 3a,b). However, mice did maintain similar overall running speeds in active and passive listening contexts (Extended Data Figure 3c,d). In a third context, photostimuli were delivered without sounds (Extended Data Figure 6).

We expected that PPC responses to the relevant sound cue would be enhanced in the active listening context, based on the effects of task engagement on visual responses in PPC [10]. Indeed, the trial-averaged responses of all cell types appeared stronger in active than passive listening (Figure 2d, Extended Data Figure 5a–b). To quantify sound-evoked responses within each context, we computed a sound modulation index (SMI, see Methods Section 0.10), comparing activity pre- and post-sound stimulus onset (Figure 2e). Neurons could have either positive or negative SMIs (Figure 2f); therefore we took the absolute value of the SMI to compare active and passive sound responses. The absolute SMI was higher in active than passive listening for all cell types [including only neurons that were sound-responsive in active, passive, or both contexts, see Methods Section 0.10; Pyr *p* < 0.0001, SOM *p* < 0.0001, PV *p* < 0.0001, paired permutation test, Figure 2g; Extended Table 2. Using the full neural population rather than just significantly sound-driven neurons yielded similar results, Extended Data Figure 7].

**Extended Data Figure 4:**
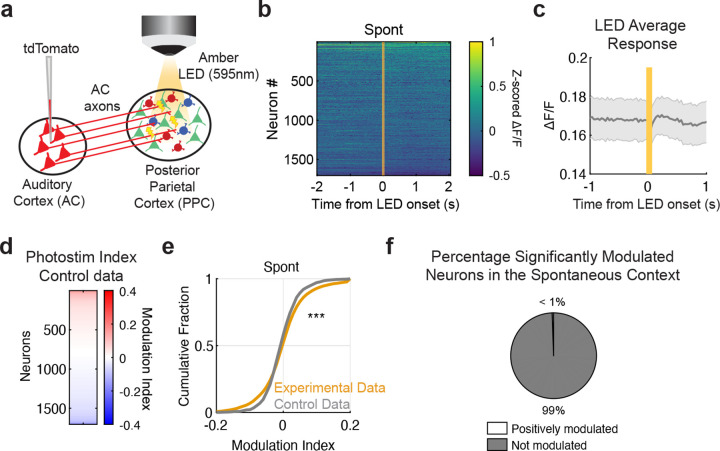
**, related to** Figure 2 Illumination of auditory axons in control mice expressing tdTomato instead of ChrimsonR does not elicit responses. (a) As a control for photostimulation, syn-tdTomato virus was injected in the auditory cortex (AC). Amber LED was used to provide fullfield illumination of AC axons in PPC for 100 ms at 7.8–11.7 mW. (b) Trial-averaged activity heatmap aligned to LED onset during the spontaneous context of all recorded neurons (*n* = 1705). (c) Average response of all neurons during the spontaneous context (shading: s.e.m. across datasets; data from 3 mice, 8 imaging sessions). (d) Heatmap of the photostimulation modulation indices across all recorded neurons in the control experiment (*n* = 1705). (e) Distribution of photostimulation modulation index in all neurons from control and experimental datasets (unpaired permutation test, *p* = 7.9992 × 10^−4^; *n*_control_ = 1705 neurons from 8 datasets; *n*_experimental_ = 7545 neurons from 24 datasets). (f) Percentage of significantly modulated photostimulation neurons in the control experiment, averaged across datasets (*n* = 8 datasets; Positively modulated = 0.5688% ± 1.1587%, Negatively modulated = 0.0000% ± 0.0000%, Unmodulated = 99.4312% ± 1.1587%; mean % ± s.d.).

We next considered whether all auditory inputs in PPC are enhanced with engagement in the auditory task, or if this effect is selective to only the learned, task-relevant stimuli. To test this, we compared the responses of neurons to AC axon photostimulation (a task-irrelevant ‘sensory’ input to PPC, see Extended Data Figure 6) in active and passive listening contexts. In contrast to the task-relevant sound response, the photostimulus responses appeared weaker in the active listening context in all cell types (Figure 2h). We computed a photostimulus modulation index (PMI) for each neuron to quantify the effects of the axon photostimulation on its activity (See Methods Section 0.10, Figure 2i). Control mice that did not express the opsin showed no systematic modulation of activity by photostimulation (Extended Data Figure 4). In contrast to the enhancing effect on relevant sound responses, photostimulus responses were weaker in all three cell types in active listening (Pyr *p* = 0.0011, SOM *p* = 0.0081, PV *p* = 0.0059, paired permutation test, Figure 2j–k, only neurons with significant PMIs in the spontaneous context were included, see Methods Section 0.10 and Extended Data Figure 7 shows similar effects when using all neurons). Together, these findings suggest that during active listening, responses to task-relevant sensory inputs are selectively enhanced in PPC activity, while responses to behaviorally irrelevant inputs are dampened. The effects were consistent across Pyr, SOM, and PV neurons, indicating a shared context-dependent modulation across cell types.

We next related the effects of behavioral context on sound and photostimulus-evoked activity. Approximately one quarter of neurons responded to sounds, and these neurons were not affected by the AC axon photostimulus (purple section in Figure 2l). A smaller fraction of neurons responded to the photostimulus and not sounds (4% of all neurons, orange in Figure 2l), while the smallest fraction was responsive to both sounds and photostimuli (cyan, Figure 2l). These results indicate that sounds and photostimuli predominantly engaged distinct subpopulations in PPC, with limited overlap.

We then examined how post-stimulus sound-evoked and photostimulation-related responses were related within these functional classes, by comparing each neuron’s average sound-evoked and photostimulation-related responses (ΔStim= post sound and photostimulus response - post sound only response). In both active and passive contexts, the activity of the most sound-responsive neurons was suppressed when photostimulation was also present (ΔStim < 0, purple circles with large post sound responses are below *y* = 0 in Figure 2m–n). In contrast, photostimulation-responsive neurons (ΔStim > 0) had little evoked activity when sounds were presented alone (yellow and cyan circles in Figure 2m–n). Consistent with this pattern, correlating sound-evoked responses with ΔStim revealed a significant negative relationship for sound-responsive neurons in both active (−0.349 ± 0.090, *p* = 0.0018) and passive (−0.345 ± 0.062, *p* = 1 × 10^−4^) contexts (unpaired permutation test against zero; Figure 2o), indicating that neurons with stronger sound responses were more strongly suppressed when photostimulation was present. These findings suggest that task engagement may alter inhibition between sound- and photostimulation-responsive neurons, favoring task-relevant activity over task-irrelevant inputs.

### Pre-stimulus activity in PPC predicted sensory responses and behavioral performance

**Figure 3.**
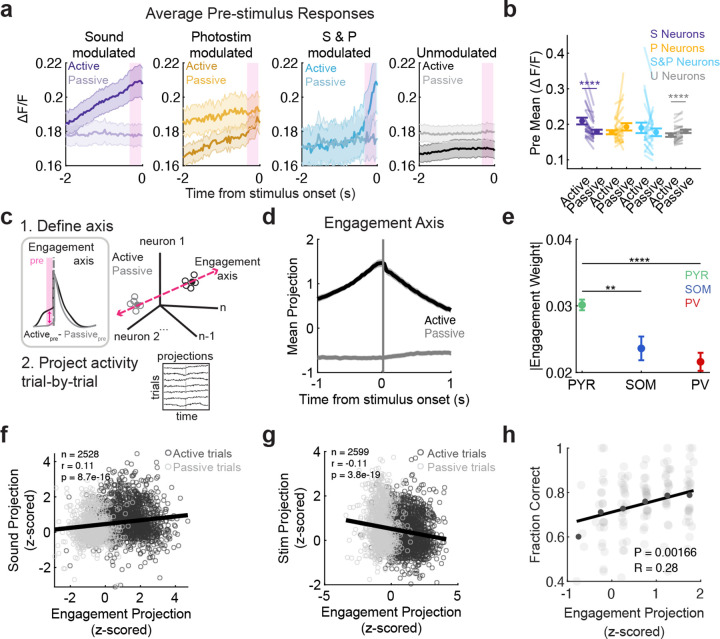
Pre-stimulus engagement predicted trial-by-trial sound and photostimulation responses and behavioral performance. (a) Trial-averaged pre-stimulus activity of all sound-responsive, photostimulation-responsive, sound and photostimulation-responsive, and unmodulated neurons in active (dark) and passive (light) contexts (mean ± s.e.m. across datasets, sound-responsive: *n* = 24; Photostimulation-responsive: *n* = 24; Sound & Photostimulation-responsive: *n* = 23; Unmodulated: *n* = 24). Pink rectangle highlights the pre-stimulus window used in the analyses. (b) Pre-stimulus Δ*F/F* of sound-responsive (S), photostimulation-responsive (P), both (S & P), and unmodulated (U) neurons across contexts (lines = within-session comparisons; mean ± s.e.m. across datasets; Sound-responsive: *p* < 0.0001, *n* = 24; Photostimulation-responsive: *p* = 0.0980, *n* = 24; Sound & Photostimulation-responsive: *p* = 0.2645, *n* = 23; Unmodulated: *p* < 0.0001, *n* = 24; paired permutation test). (c) Schematic of trial-by-trial population activity projection analysis. Axes were defined by the mean difference in pre-stimulus population activity between contexts (active – passive). Each trial’s population activity (neurons – time) was projected onto these axes, yielding a projection time series for each trial. (d) Mean trial-aligned projections onto the engagement axis in active (black) and passive (gray) contexts (mean ± s.e.m. across datasets, *n* = 24). (e) Contribution of each cell type to the engagement axis (absolute value of the projection weight; mean ± s.e.m. across datasets; paired permutation test: *p*_pyr–som_ = 0.0017, *p*_pyr–pv_ < 0.0001, *p*_som–pv_ = 0.2941; *n* = 24). (f–g) Relationship between pre-stimulus engagement projection and post-stimulus sound or photostimulation projections across trials. Black lines represent the fitted slope from a linear regression model; each dot represents a trial. (h) Behavioral performance (fraction correct) as a function of binned pre-stimulus engagement projection on active trials, bin counts: *n* = 4, *n* = 21, *n* = 24, *n* = 24, *n* = 24, *n* = 24. Gray dots indicate individual datasets, black dots indicate mean across datasets, and black line is the fitted slope from a linear regression model. For all panels, n.s. means no significance; *, *p* < 0.05; **, *p* < 0.01; ***, *p* < 0.001; ****, *p* < 0.0001.

We hypothesized that behavioral contexts such as active and passive listening shift PPC into different population activity states that determine its responses to incoming sensory signals. For example, the activity of task-relevant sound- and task-irrelevant photostimulus-driven neurons could be differentially modulated by context, even before the stimuli are presented. To test this, we examined the trial-averaged pre-stimulus activity in the significantly sound-responsive neurons, photostimulus-responsive neurons, and neurons not responding to either (‘Unmodulated’) (Figure 3a–b; Extended Table 3). Pre-stimulus activity was significantly higher in sound-responsive neurons in active listening than passive listening (sound-responsive: *p* < 0.0001, photostimulation-responsive: *p* = 0.0980, sound & photostimulation-responsive (S & P) : *p* = 0.2645, unmodulated: *p* < 0.0001; paired permutation test, Figure 3a–b). In contrast, pre-stimulus activity in the active context was lower in neurons that did not respond to photostimuli or sounds (unmodulated *p* = 0.0001, Figure 3a–b). To further quantify the effects of context on pre-stimulus activity, we calculated an engagement index (EI) for each neuron (see 0.10. Positive EIs reflect higher activity in the active context, while negative EIs reflect higher activity in the passive context. In general, EI values tended to be negative (Figure 8c) except in sound-responsive neurons, which had positive EI values (Extended Data Figure 8g). These results indicate that behavioral engagement selectively enhanced baseline activity in neurons encoding behaviorally relevant information in PPC.

To assess how engagement modulates responses to the stimuli themselves, we next tested whether pre-stimulus activity patterns could predict the population’s responses to the task-relevant sounds and task-irrelevant photostimuli. To capture the trial-to-trial variation in the population’s modulation by ‘engagement’ level, we found the axis in high dimensional population activity space that best aligned with the difference in pre-stimulus activity between the active and passive contexts (Figure 3c–d), and projected each trial’s population activity onto this axis (See Methods Section 0.11). To determine relative contribution of Pyr, PV, and SOM neurons in defining the engagement axis, we examined the distribution of engagement axis weights by cell type. The absolute values of the weights were significantly larger in Pyr than in SOM or PV neurons (*p*_pyr-som_ = 0.0017; *p*_pyr-pv_ < 0.0001; *p*_som-pv_ = 0.2941; unpaired permutation test; Figure 3e), indicating that fluctuations in engagement are predominantly reflected by the excitatory population. When we examined the raw engagement weights, we did not observe significant differences across cell types (Kruskal-Wallis, *p* = 0.8522; Extended Data Figure 9f), although SOM neurons tended to have positive weights in the engagement axis, while PV and Pyr neurons tended to have negative weights (Extended Data Figure 8e).

We also defined sound- and photostimulation-related population activity axes based on the corresponding population responses (numerators of the modulation indices in Figure 2e,i), allowing us to then assess how pre-stimulus activity along the engagement axis related to subsequent sound and photostimulusrelated population responses (Extended Data Figure 9a–b). We correlated the trial-to-trial fluctuations in population activity along the engagement axis in the pre-stimulus period to the population’s activity along the sound and photostimulus axes. Pre-stimulus population engagement was positively correlated to the population’s response to sound stimuli (Pearson’s correlation *r* = 0.16, *p* = 8.7e-16, Figure 3f), while it was negatively correlated to the population’s response to the task-irrelevant photostimulus (Pearson’s correlation *r* = −0.17, *p* = 3.8e-19, Figure 3g). Engagement axes constructed from only PV or SOM neurons predicted stimulus-evoked responses with similar trends as the full-population axis (Extended Data Figure 9e), suggesting that engagement state modulates activity in all cell types. Thus, the pre-stimulus population activity pattern predicted both the enhancement of the population’s responses to sounds and the suppression of its responses to photostimuli in the active context.

While these effects were dominated by larger differences between active and passive listening (comparing light and dark dots in Figure 3f–g), we observed smaller shifts in the engagement-related activity even within contexts (Figure 2f–g, Extended Data Figure 9). We wondered whether pre-stimulus population activity pattern was also related to the mouse’s trial-to-trial engagement in the task and therefore behavioral performance accuracy. Indeed, the pre-stimulus activity pattern was positively correlated with behavioral performance (Pearson’s *r* = 0.28, *p* = 0.00166, Figure 3h). Together, our results suggest that task engagement primes the PPC population to selectively enhance responses to behaviorally relevant stimuli, while quenching responses to irrelevant stimuli. Furthermore, these effects are predictive of the mouse’s behavioral accuracy in responding to these sensory cues.

### Selective contextual inputs enhance sound responses and suppress photostimulation responses in a network model

**Figure 4.**
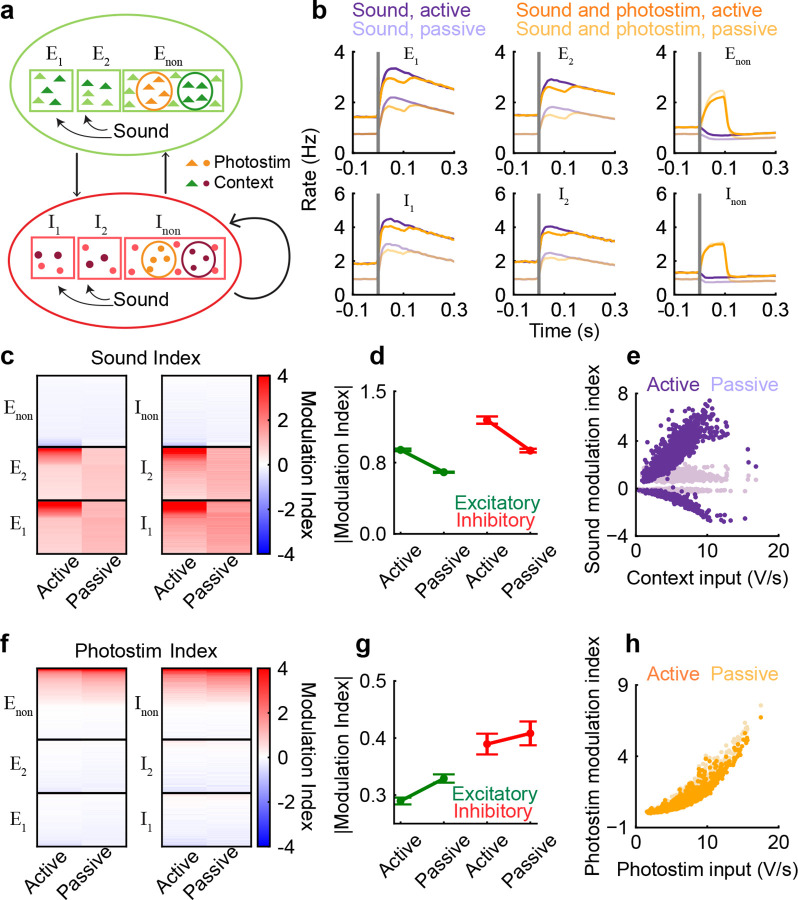
Selective contextual inputs enhance sound responses and suppress photostimulation responses in a network model. (a) Model schematic. The model consisted of three excitatory subpopulations (*E*_1_*, E*_2_ and *E*_non_) and three inhibitory subpopulations (*I*_1_*, I*_2_ and *I*_non_). The *E*_1_, *E*_2_, *I*_1_ and *I*_2_ neurons received sound inputs, with *E*_1_ and *I*_1_ neurons receiving larger input during left sound and *E*_2_ and *I*_2_ neurons receiving larger input during right sound. A subset of *E*_non_ and *I*_non_ neurons received photostimulation inputs (orange triangles in *E*_non_ and orange dots in *I*_non_). Context inputs were applied to a set of randomly selected neurons in all populations that did not receive photostimulation inputs (neurons with darker shades in *E*_non_ and *I*_non_) throughout active listening trials. The excitatory and inhibitory neurons were randomly connected without a structure specific to the subpopulations. There was no connection among the excitatory neurons. (b) Population-averaged firing rates with only sound inputs (purple) or sound and photostimulation inputs (orange), in passive (lighter) or active (darker) listening contexts. The sound inputs to *E*_1_ and *I*_1_ neurons were larger than those to *E*_2_ and *I*_2_ neurons. The onset time of both sound and photostimulation inputs was at *t* = 0 (gray line). The sound input lasted for 500 ms and the photostimulation input lasted for 100 ms. (c) Heatmaps for the sound modulation index for excitatory (left) and inhibitory (right) populations. (d) The average absolute value of the sound modulation indices of both the excitatory (green) and inhibitory (red) neurons were larger in the active context, consistent with data (Figure 3f,g). The change of the average absolute values of sound modulation indices from the passive to active context is 0.23 for excitatory and 0.32 for inhibitory neurons. (e) Active (dark purple) and passive (light purple) sound modulation index as a function of context input for the neurons which received context inputs in the active context. Neurons that received larger context inputs were either more activated or more suppressed by sounds. (f, g) Same as (c, d) for the photostimulation modulation indices. The average magnitudes of photostimulation modulation indices were lower in the active context for both the excitatory and inhibitory neurons, consistent with data (Figure 3j,k). The change of the average absolute values of photostimulus modulation indices from the passive to active context is −0.039 for excitatory and −0.019 for inhibitory neurons. (h) Active (dark orange) and passive (light orange) photostim modulation index for the neurons which received photostimulation inputs. The photostim modulation indices increased with the strength of the photostimulation input and the photostim modulation index12 in the active context was lower than that in the passive context across input strengths.

In order to understand the mechanisms underlying the selective filtering of auditory signals in the active listening context (Figure 2, 3), we constructed a network model of randomly connected excitatory and inhibitory neuron populations (Figure 4a). Each population consisted of three subpopulations based on their responses and selectivity to sound location. The three excitatory subpopulations were: left sound selective (*E*_1_), right sound selective (*E*_2_) and non-selective (*E*_non_) subpopulations. Similarly, the inhibitory subpopulations were left sound selective (*I*_1_), right sound selective (*I*_2_) and non-selective (*I*_non_) subpopulations. The connectivity among neurons only depended on their cell types (*E* or *I*) and was not specific to the subpopulations (see Methods section 0.16). Since the connection probability between excitatory neurons was found to be very low in PPC [8], we assumed there were no connections between excitatory neurons in the model for simplicity.

The subpopulations of excitatory and inhibitory neurons mainly differed in the type of inputs they received. The sound inputs were applied to *E*_1_*, E*_2_*, I*_1_ and *I*_2_ neurons. *E*_1_ and *I*_1_ neurons received more inputs from left sounds, while *E*_2_ and *I*_2_ neurons received more inputs from right sounds. *E*_non_ and *I*_non_ neurons did not receive sound inputs. Since our experimental results suggested that neurons that were positively modulated by photostimulation did not respond strongly to sound (Figure 2m,n), we assumed that the photostimulation inputs were only applied to subsets of *E*_non_ and *I*_non_ neurons in the model (Figure 4a, orange). In the active listening context, additional ‘context’ inputs were applied to a random set of neurons from all populations including *E*_non_ and *I*_non_ (Figure 4a, neurons with darker shade); however, no neuron received both photostimulation and context inputs. The context inputs model the enhancement of pre-stimulus activity in sound neurons in the active listening context (Figure 3a,b).

We simulated the presentation of sounds and photostimuli in the model, mimicking the presentation to mice *in vivo*. The average firing rates of *E*_1_, *E*_2_, *I*_1_ and *I*_2_ populations increased after sound onset (Figure 4b, purple) and all neurons in these four subpopulations had positive sound modulation indices (Figure 4c). In contrast, the *E*_non_ and *I*_non_ neurons were suppressed by sounds, resulting in negative sound modulation indices. This was due to the elevated inhibition arriving from *I*_1_ and *I*_2_ (Figure 4b,c). The presentation of photostimulation with sounds activated the *E*_non_ and *I*_non_ neurons and slightly suppressed the responses of *E*_1_, *E*_2_, *I*_1_ and *I*_2_ neurons (Figure 4b, orange vs purple). These results demonstrate the mutual inhibition between sound-responsive and photostim-responsive neurons in the model, which is mediated by the non-specific recurrent inhibition.

In the active context, a random set of neurons were depolarized by the context inputs, which increased their pre-stimulus firing rates. Because of the supralinearity of neurons’ input-output transfer function, an increase in the pre-stimulus rate resulted in a higher response gain to sound input. On the one hand, *E*_1_, *E*_2_, *I*_1_ and *I*_2_ neurons that received context inputs responded more strongly to sound with larger modulation indices (Figure 4c–e). On the other hand, *E*_non_ and *I*_non_ neurons that received context inputs became more suppressed during sound with more negative modulation indices (Figure 4c–e). Therefore, the average magnitude of sound modulation was much larger in the active context than in the passive context.

The enhanced responses of the *I*_1_ and *I*_2_ neurons to sound in the active context results in more inhibition in *E*_non_ and *I*_non_ neurons that received photostimulation inputs. As a result, their responses to photostimulation were suppressed in the active context (Figure 4f–h). The segregated targeting of context and photostimulation inputs is important for the suppression of photostimulation responses. When context inputs were allowed to randomly target neurons that received photostimulation input, the average magnitude of photostim-modulation indices was enhanced, rather than suppressed, in the active context (Extended Data Figure 10c). This is because the neurons that received both context and photostimulation inputs had much larger photostim-modulation indices in the active context (Extended Data Figure 10d). A comparison of the two cases (i.e. with segregated and overlapping targets) yields a p-value of 2 × 10^−4^ for the active excitatory photostim modulation index and 0.003 for the inhibitory photostim modulation index. The small p-values indicate that the reversal of the trend between active and passive photostim modulation index in the second case, i.e. with overlapping photostimulation and contextual targets is statistically significant and cannot be explained by network noise alone.

In summary, we find that sound-responsive and photostim-responsive neurons mutually inhibit each other through non-specific recurrent inhibition in our model. Context inputs selectively activate some of the sound neurons and increase their responses to sound due to the supralinearity of their input-output transfer functions. The enhanced activity in sound-responsive neurons in turn suppresses the activity in photostim-responsive neurons. We find that tuning-specific structure in connectivity is not required to explain our experimental findings. Recent work from mouse PPC area suggests an opponent inhibition motif in connectivity between neurons with different choice selectivity [8]. Incorporating such a connectivity motif in our model produces similar results, though to a smaller degree in the context-regulated changes in the sound modulation (Extended Data Figure 10e–h).

## Discussion

Together, our results identify a potential circuit mechanism allowing for selective gating of sensory responses in parietal cortex. Even before task-related stimuli were delivered, neural activity was modulated up or down depending on the behavioral relevance of response preferences. Activity was increased specifically in neurons responsive to task-relevant sound cues, while neurons lacking task stimulus-related responses were suppressed. Strikingly, the strength of activity modulation before the sensory stimuli were even delivered could predict the mouse’s behavioral accuracy on that trial in the task. This indicates that task engagement establishes a structured pre-stimulus population activity state that selectively enhances the response gain of behaviorally informative neurons.

We built network models with different connectivity schemes, and revealed that the selective enhancement of task-relevant responses that we observed *in vivo* requires a selective depolarizing input to these neurons in the model. This could be accomplished through a number of different biologically plausible circuit mechanisms. For example, plasticity-dependent changes in synaptic strength could allow top-down projections from higher cortical areas to more strongly target relevant sound encoding neurons [30, 31]. Alternatively, convergent inputs from multiple areas such as neuromodulatory centers, thalamic inputs, and higher cortical areas [32, 33] could lead to the selective depolarization of behaviorally relevant neurons. Notably, random local inhibitory connectivity was sufficient to drive the quenching of irrelevant inputs, and specific lateral inhibition between sound and photostimulation responding neurons was not required. Still, this could be provided by a specific inhibitory cell class, such as SOM neurons. These findings contrast but do not necessarily conflict with prior studies emphasizing the structured inhibitory interactions during decision-related computations [7, 8]. While choice-related PPC representations may rely on opposing inhibitory motifs, the selection of behaviorally relevant sensory inputs for integration can arise in more unstructured local networks.

PV and SOM neurons did not differ greatly from each other in the task-related information they encoded (Figure 1), or in their activity modulation with behavioral context (Figure 2). Instead, the feature that best distinguished cell types in our study was the temporal organization of activity throughout the trial. The temporal organization of activity across cell types may reflect how differently balanced inhibitory dynamics are implemented in time. SOM neurons showed sustained activity during the intertrial interval, while Pyr and PV were more active during task execution. These temporal differences could provide a mechanism for stabilizing population dynamics between behavioral epochs, helping to maintain the flexible integration of sensory inputs in association cortex. Future experiments targeting SOM neurons during intertrial periods will be critical to test whether their activity shapes population readiness or transitions between task states.

PV and SOM neurons themselves are not homogeneous inhibitory cell classes, but instead can be further subdivided based on morphology, firing patterns, and protein expression patterns [15, 34, 35]. Recent studies have uncovered different roles for these more specifically defined cell classes. For example, calbindin-2 expressing SOM neurons have different learning-related plasticity of sensory responses than the general SOM population [36]. Another genetically-defined subpopulation that includes calbindin-2 neurons is gap-junction coupled, and responds in PPC specifically during course corrections in VR [37]. The SOM neurons in our study had a diverse range of task variable encoding, as well as engagement-related activity (Extended Data Figure 8e). Engagement trended in a different direction than other cell types, with overall positive effects of task engagement on baseline activity in SOM neurons. A more refined genetic approach to target specific subclasses of SOM neurons will be necessary to determine the contributions of SOM subclasses to these distributions.

## Methods

### 0.1 Animals

All procedures were approved by the University of Pittsburgh Institutional Animal Care and Use Committee. Homozygous SOM-Flp mice (Cat no. 31629, Jackson Laboratory, USA) were crossed with homozygous PV-Cre mice (Cat no. 17320, Jackson Laboratory, USA) at Charles Rivers Laboratories. All experiments were conducted using the F1 generation, which expressed Flp in SOM+ neurons and Cre in PV+ neurons. Mice were group housed in cages with between 2 and 5 mice. Adult (8–40 weeks) male (n = 3) and female (n = 3) mice were used for the main experiments. Additional mice (2 males) were used as optogenetic controls. All mice were maintained on a reversed 12-hour light/dark cycle, and all experiments were carried out during the dark (active) phase.

### 0.2 Surgery

Mice were anesthetized with isoflurane (4% for induction, and 1–2% maintenance during surgery), and mounted on a stereotaxic frame (David Kopf Instruments, CA). Dexamethasone was injected 12–24 hours prior to surgery, and carprofen and dexamethasone (Covetrus, ME) were injected subcutaneously immediately prior to surgery for pain management and to reduce the inflammatory response. The eyes were covered with ophthalmic ointment (Henry Schein, NY). Nair was applied to remove hair from the top of the head. Prior to making the incision, lidocaine was injected subcutaneously into the scalp. A 2 mm craniotomy was made over left AC (centered at 3.0 mm posterior and 4.3 mm lateral to bregma) using a biopsy punch (Integra^™^ Miltex^™^ Standard Biopsy Punches) and a 3 mm craniotomy was made over left PPC (centered at 2mm posterior and 1.75mm lateral to bregma) using a hand drill. A 1:1:2 mixture of three viruses (1, Addgene no. 114471 AAV1-Ef1a-fDIO-mCherry, 2, Addene no. 28306 AAV1-FLEX-tdTomato, and 3, Addgene no. 100837 AAV1.Syn.GCaMP6f.WPRE.SV40A) was loaded into the glass injection pipet and injected into PPC. A 1:1 mixture of two viruses (1, GCamp6f and 2, Addgene no. 59171, AAV1-Syn-ChrimsonR-tdTomato) was injected into AC. A micromanipulator (QUAD, Sutter, CA) was used to guide the pipette containing virus mixture to be 250μm under the dura at each site, where 60nL virus was pressure-injected over 5–10 minutes. Pipettes were maintained in place for 5 minutes post-injection to prevent backflow. Dental cement (Parkell, NY) was mixed with black India ink (Dr. Ph. Martin’s Bombay India Ink) to create a black cement mix. The black ink was used to minimize light reflection from the virtual reality maze and during photostimulation. The black cement ink was used to seal a glass coverslip (4mm for PPC, 3mm for AC) over a drop of Kwik Sil (World Precision Instruments, FL) over the craniotomy. Using dental cement, a one-sided titanium headplate was attached to the right hemisphere of the skull. After mice had recovered from the anesthesia, they were returned to their home cages and received oral carprofen tablets after surgery and for 3 days post-surgery (Fisher Scientific, MA). Control mice surgeries included the methods described here except that a mixture of 1:1 mixture of GCamp6f (Addgene no. 100837) and AAV1-CAG-tdTomato (Addgene no. 59462) was injected into AC.

### 0.3 Behavioral task

Mice were placed on water restriction one week before starting behavioral training around 9–10 weeks of age, at least 1 week after undergoing surgery.

Mice were trained on a navigation-based sound localization task as previously described [25]. Mice ran down the stem of a virtual reality (VR) T-maze and judged the location of the sound cue, determining whether it came from the left (-90 degrees) or right (+90 degrees) speaker. They reported their decisions by turning into the corresponding arm of the T-maze to receive a water reward (4μl per reward). The maze’s stem featured black and white stripes against a gray wall to provide visual flow, while the back wall had white polka dots on a black background to help distinguish the arms from the stem.

The sound stimuli were dynamic ripples (1s duration), starting at a specific position in the maze (32 cm from starting position) and repeating after 250ms until the mouse turned into the left or right arm at the end of the stem. Upon crossing a spatial threshold into one arm or the other, a 250ms ‘reward tone’ accompanied the water reward if the trial was correct, while a 400ms ‘incorrect tone’ played at the point the mouse would have received a reward had the trial been correct. All sounds were calibrated to play at 70dB. The reward period was followed by a 3 second or 5 second inter-trial-interval (ITI) for correct and incorrect trials respectively. During the ITI, the virtual reality screens turned gray to maintain consistent mean luminance between the maze and ITI epochs of trials. Mice performed 200–300 trials during each behavioral session.

### 0.4 Two-photon microscope

Images were acquired using a resonant scanning two-photon microscope (Ultima Investigator, Bruker, WI) at a 30 Hz frame rate and 512 × 512 pixel resolution through a 16x water immersion lens (Nikon CF175 16X/0.80 WD = 3 mm). PPC was imaged at a depth between 150 *μ*m and 250 *μ*m which corresponds to cortical layer 2/3. The angle of the objective was matched to the angle of the window (≈ 10degrees). Excitation light was provided by both a tunable femtosecond infrared source (780–1100nm) and a fixed 1045nm wavelength laser (Insight X3, Spectra-Physics, CA). Tunable and fixed wavelength beams were combined with a dichroic mirror (ZT1040dcrb-UF3, Chroma, VT) before being routed to the microscope’s galvanometers. Green and red wavelengths were separated through a 565nm lowpass filter before passing through bandpass filters (Chroma, ET525/70 and ET 595/50, VT). PrairieView software (v5.5 and v5.7 Bruker, WI) was used to control the microscope. For functional imaging the microscope was tuned to 920nm.

### 0.5 Imaging protocol

Imaging began 3–5 weeks post-surgery once robust expression of the GCaMP6f virus was confirmed. Fields of view were selected based on co-expression of viral GCaMP6f (all neurons) and red fluorophores (PV and SOM neurons). Multiple imaging sessions were conducted for each cranial window, focusing slightly different depths and lateral/posterior locations within the imaging windows across sessions. PPC was imaged in 6 mice with each window imaged up to 6 times. Imaging from a cranial window was suspended when we observed nuclear inclusion in 3 or more cells in the field of view, indicating an over-expression of GCaMP6f. During each imaging session, GCaMP6f fluorescence changes were imaged in Pyramidal, SOM (mCherry+), and PV (tdTomato+) neurons, while mice ran on a spherical treadmill. A black balloon encompassing the objective was lowered onto the outside of the black cement well to prevent any light from entering or exiting the field of view. Each session lasted between 60–120 minutes, with 60 minutes in the active task context, 30 minutes in passive listening, and 30 minutes in spontaneous context.

Each imaging session started with the mice performing the behavioral task (active listening, see Methods Section 0.3). In one-third of the trials, the onset of the first sound repeat was paired with AC axon photostimulation (see Methods Section 0.6). Another one third of trials were controls with no photostimulation (0mW), while the final one third of trials had no manipulations during sound onset. Both the control and no manipulation trials were considered “sound only” trials.

Following the behavioral task, mice were exposed to two additional behavioral contexts, during which imaging continued for approximately 30–60 minutes. The VR monitors displayed a constant gray screen, and the reward spout was removed. In the passive context, the same sound stimuli from the task were presented, and one third of trials were manipulated with photostimulation similarly as in the task. In the spontaneous context, no sound stimuli were presented; instead, photostimulation was delivered at regular 10-second intervals.

### 0.6 Optogenetic stimulation of AC axons in PPC

On one third of trials, ChrimsonR expressing axons from the auditory cortex (AC) were photostimulated with an amber LED (595nm) incorporated into the two-photon microscope’s light path, at an average power of 7.8mW from the objective. The illumination source for the ChrimsonR stimulation was a switchable LED (M595L4, Thorlabs, LED driver LEDD1B) triggered with PrairieView MarkPoints. Each pulse lasted 100 ms, with a 10-second interval between pulses during the spontaneous context. In the active and passive contexts, the interval between pulses varied, depending on trial timing, as pulses were paired with the onset of the first sound repeat in each trial. Each acquisition contained multiple pulses of either stim or control (0mW) interleaved throughout the imaging session. During the photostimulation, the shutter to the photomultiplier tubes was closed, and the corresponding frames were blanked and removed, linearly interpolated with values pre- and post-photostimulation frames. A masking 595 nm LED was placed behind the VR monitors during all sessions to minimize any visual responses to the photostimulation light if any.

### 0.7 Identification of PV, SOM, and putative Pyramidal neurons

In each imaging field of view, all neurons expressed the green calcium indicator GCaMP6f, and in addition, PV neurons expressed the red fluorophore tdTomato while SOM neurons expressed the red fluorophore mCherry. We have previously established that because the excitation spectra of tdTomato and mCherry differ, we can distinguish neural populations expressing these two indicators by first acquiring images across a range of excitation wavelengths, and then clustering the fluorescence intensity of the red cells across these wavelengths as follows. Given that PV and SOM neurons constitute approximately 70% of the inhibitory neuron population, which itself represents about 10–15% of all neurons in the mouse brain, we categorized the remaining unlabeled neurons in our imaging as putative pyramidal neurons.

At the start or end of each imaging session, a wavelength series was collected from 780nm to 1100nm in increments of 20nm, averaged over 16 frames per increment across multiple powers (0–275 mW), excluding 1020 and 1060 nm wavelengths. The 1045nm image was acquired with a fixed wavelength laser. The wavelength series was processed in Suite2p, along with 1000 frames of the red channel data collected during the functional imaging phase of the same imaging session to be registered and motion corrected together. ROIs derived from Suite2p masks in the functional imaging phase that overlapped with red-channel cells were applied to the wavelength series. For each red channel ROI, mean intensity values across wavelengths were calculated and analyzed with k-means clustering to classify ROIs. The wavelength set that minimized variability across datasets (780 nm, 800 nm, 820 nm, 960 nm, and 980 nm) was chosen to classify fluorophores consistently [27]. Any ROI below the 0.7 threshold was manually curated by comparing the cluster classification to expected cluster based on their fluorescence across key wavelengths (780, 800, 980nm).

### 0.8 Image processing

For each imaging session, the raw calcium movies were processed for motion correction, cell detection, and fluorescence and neuropil extraction. These steps were performed using Suite2p 0.10.3 with Cellpose 0.7.3 integration in Python [38, 39]. After processing, ROIs were manually curated using the Suite2p GUI, to ensure that only cell bodies were included in analysis, based on morphology. A small number of red-labeled cells were manually added using the Suite2p GUI when they were clearly visible in the bleed-through corrected red channel mean image but had not been automatically detected. We scaled the neuropil fluorescence by a factor of 0.7 and subtracted this adjusted timeseries from the raw fluorescence timeseries to obtain a neuropil-corrected fluorescence signal for each selected cell.

### 0.9 ΔF/F and deconvolution

After the neuropil-corrected fluorescence was obtained, we calculated ΔF/F for each cell in each frame by calculating (F-Fbaseline)/Fbaseline for each frame, where F is the fluorescence of a given cell at that frame and Fbaseline was the eighth percentile of that cell spanning 450 frames before and after (15 seconds before and after the frame, 30 seconds total). We then deconvolved the ΔF/F timeseries to estimate the relative spike rate using the OASIS toolbox [40]. The AR1 FOOPSI algorithm was employed, with the toolbox optimizing the convolution kernel, baseline fluorescence, and noise distribution for each neuron. To filter out low-magnitude events, we applied a threshold of 0.05 a.u. on deconvolved activity. Analyses were performed using both ΔF/F and deconvolved activity, yielding consistent results.

### 0.10 Response modulation indices

To quantify how each neuron was modulated by axon photostimulation, sound stimuli, and task engagement, we calculated a modulation index for each as follows.

The Sound Modulation Index (SMI, Equation 1) was calculated by comparing activity during a 300ms window before the sound stimulus onset, with activity during the 1s window following the event. The SMI was used to evaluate how strongly a neuron responded to sound inputs alone (without photostimulation) during the active or passive contexts. The same equation was also used to calculate photostimulation responses during only the spontaneous context, when no sensory stimulus was presented.

(1) $$\text{SMI}=\frac{R_{\text{post}}-R_{\text{pre}}}{R_{\text{post}}+R_{\text{pre}}},$$

where *R*_pre_ and *R*_post_ denote the mean neural response (Δ*F/F*) across trials during the pre- and poststimulus windows, respectively.

The Photostimulus Modulation Index (PMI, Equation 2) was used to quantify the effect of AC axon photostimulation on each neuron’s sound-evoked response during the active and passive listening contexts. In these contexts, the photostimulus was applied at the time of the sound stimulus onset during photostimulus trials. In control trials, the sound stimulus but no photostimulus was applied (considered sound-only trials). These two trial types could then be compared to determine how photostimulation affected sensory responses. Activity in the 1s time window after sound + photostimulus onset was compared to activity in the 1s time window after sound stimulus onset alone (from control trials), as follows:

(2) $$\text{PMI}=\frac{R_{\text{sound}+\text{stim}}-R_{\text{sound}}}{R_{\text{sound}+\text{stim}}+R_{\text{sound}}},$$

where *R*_sound_ and *R*_sound+stim_ denote the mean neural response (Δ*F/F*) across trials during sound-only and sound+photostimulation conditions, respectively.

Trials were divided into left and right sound trials, and modulation indices (SMI and PMI) were computed separately for each side. The larger of the two was taken as the modulation index for each neuron.

The Engagement Index (EI, Equation 3) was used to quantify the effects of behavioral state in the pre-stimulus period. Activity in the 300 ms time window before stimulus onset in the active context was compared to activity in the 300 ms time window before stimulus onset in the passive context, as follows:

(3) $$\text{EI}=\frac{R_{\text{active},\;\text{pre}}-R_{\text{passive},\;\text{pre}}}{R_{\text{active},\;\text{pre}}+R_{\text{passive},\;\text{pre}}},$$

where *R*_active, pre_ and *R*_passive, pre_ denote the mean neural response (Δ*F/F*) across trials.

Neurons were classified as significantly sound-modulated if the sound-evoked response in at least one context (active, passive, or both contexts) was significant. Neurons were classified as significantly photostim-modulated if the photostimulus-evoked response in the spontaneous context was significant. Significance was determined via a bootstrapping approach. Specifically, we randomly shuffled the labels of the average responses being compared (pre vs. post for sound modulation, stim vs. stim+sound for photostimulation modulation, active pre vs. passive pre for engagement modulation) 1000 times and recalculated the modulation index in Equations 1, 2, 3 for each shift. Neurons with true modulation indices greater than 97.5% or less than 2.5% of the bootstrapped distribution, and with modulation indices exceeding a threshold of above 0.1 or below −0.1, were considered significantly positively modulated or significantly negatively modulated.

### 0.11 Population analyses

To characterize the patterns of the population activity related to task engagement, sound stimuli, and photostimulation, we found the axes in population activity space that best described the population’s response to these variables. In a population of *n* neurons recorded simultaneously, we calculated each axis by calculating the difference between the mean *n*-dimensional vector between two sets of responses. For the sound axis, we used only sound only trials, and compared the mean vectors in the pre- and post-sound stimulus onset windows. For the photostim axis, we took the difference of mean post stimulusonset responses during photostim + sound trials and the mean response during sound only trials. For the engagement axis, we took the differences in the mean pre-stimulus period between active and passive listening contexts. The resulting difference is a weight vector of size (*n* × 1). This weight vector was then normalized by its *L*_2_-norm. Population activity was then projected onto each axis using held-out test trials, by computing the dot product between the weight vector and the matrix of neural activity for each trial (frames × *n*), yielding a projection time series per trial. To avoid overfitting, we performed 4-fold cross-validation: the full set of trials was partitioned into four non-overlapping folds, with three folds (75%) used for training axis weights and the remaining fold (25%) reserved for testing. This procedure was repeated four times such that each trial served as a test trial exactly once. Projections were always computed on held-out test trials, and all test trials across folds were concatenated for final analyses (Figure 3).

### 0.12 Population decoding

A linear support vector machine (SVM, MATLAB; fitcsvm, box constraint = 1) was trained on each time bin of the population activity in each session (using non-overlapping 90 ms time bins) to decode sound category (left vs. right sound), choice (left vs. right turn), and outcome (correct vs. incorrect). We used 10-fold cross-validation within the training dataset (70% of trials), structuring folds to maintain a balanced distribution of trials across all stimulus, choice, and outcome combinations. The data were aligned by the onset of each of three sound repeats, the mouse’s turning onset into one of the two maze arms, and reward delivery onset in correct trials (or incorrect tone for error trials). To account for temporal variability across sessions, we identified the minimum number of imaging frames between trial start and first sound onset, third sound offset and turn onset, and turn onset and reward onset, and truncated all trials accordingly. Neural data was z-scored before classifying it.

To mitigate potential confounds and ensure that variables were decorrelated for decoding, we performed random subsampling of trials. For sound category and choice decoding, we balanced the data by including equal numbers of trials for each combination of sound stimulus and choice. For outcome decoding, the same approach was applied to decorrelate choice and outcome. Trials were selected such that each combination of conditions was equally represented, with a minimum of four trials per condition. Datasets without sufficient trials were excluded from decoding the affected variable. In total, 2 of 25 datasets were excluded from sound category and choice decoding, and 6 of 25 from outcome decoding. This random subsampling was repeated 50 times. Decoder accuracy was evaluated on the held-out test data for each fold.

Neural populations were grouped in multiple ways: (1) full Pyr, SOM, or PV populations, (2) all recorded neurons, (3) randomly subsampling neurons to match the lowest cell type count, and (4) topranked Pyr neurons based on their information content, also matching the lowest cell type count. For summary analysis, mean decoding accuracy was first computed across folds, then averaged across datasets in a 450 ms window following the onset of the relevant event: the first sound repeat (S1) for sound category decoding, turn initiation (T) for choice decoding, and reward delivery (R) for outcome decoding.

### 0.13 Single neuron decoding

To estimate the information represented in individual PPC neurons about sound category, choice, and outcome, we constructed a probabilistic decoder to classify these variables on single trials based on each neuron’s activity. The decoder methodology follows the approach described in [25]. Specifically, for each trial, we decoded one of the three task variables by calculating the probability of each condition given the recorded neural activity. The decoded variables included: sound category (left vs. right sound), choice (left vs. right turn), and outcome (correct vs. incorrect). Data partitioning, variable decorrelation, dataset elimination based on minimum trial numbers, and trial alignment followed the same approach as in population decoding (0.12).

The decoding process employed Bayes’ theorem, leveraging neuron response probabilities estimated using an encoding GLM [25]. For each trial, the GLM provided the conditional probabilities of neural responses based on predictors relevant to that trial. Using these probabilities, we computed the posterior probability of each possible condition.

The decoder operated on an instantaneous timescale, using neural activity from a single imaging frame (~30 ms) at each time point *t*. For each time point, the decoded condition was defined as the one with the maximum posterior probability, derived from either individual neuron responses or the combined activity of the simultaneously imaged neuronal population.

To isolate true sensory encoding, peak information content for sound category was extracted from a stimulus-locked window spanning the three auditory stimuli presented early in the trial (S1 onset to S3 offset). Restricting the analysis to this sensory epoch prevented contamination from later choice-, movement-, and reward-related activity, which can artificially inflate sound decoding accuracy even under balanced trial sampling. In contrast, peak information for choice and outcome was computed over the full trial duration, as these variables evolve throughout the decision and reward epochs. Neurons were considered informative if their peak information was above 0.06 bits [25].

### 0.14 Statistics

All pairwise comparisons were assessed using two-sided paired or unpaired permutation (i.e. randomization) tests with 10,000 iterations. A p value of < 0.0001 reflects the smallest possible value given the number of permutations performed. All permutation tests were performed for differences in means. For statistical comparisons involving more than two groups, we used Kruskal-Wallis (non- parametric ANOVA) and used paired or unpaired permutation tests post-hoc to determine which groups differed from each other. Data were naturally grouped by (1) context (active or passive) and (2) cell type (Pyr, SOM, or PV) or functional groups (Sound, Photostim, Sound & Photostim, Unmodulated). Values are presented as mean ± s.e.m. unless otherwise stated. Box plots indicate the median (center line), 25 and 75 quartiles (box limits) and the most extreme data points within 1.5× the interquartile range from the quartiles (whiskers). Bonferroni correction was applied for multiple comparisons. All analyses were performed in Python or MATLAB.

### 0.15 Histology

After all imaging sessions had been acquired, each mouse was transcardially perfused with 1X PBS and then 4% paraformaldehyde. The brain was extracted, cryoprotected, embedded, frozen, and sliced using a cryostat. Once slide mounted, we stained brains with DAPI to be able to identify brain structures. We used anatomical structure to verify the locations of our injections in PPC and AC.

### 0.16 Network model

The network model consisted of 8000 neurons divided into three excitatory and three inhibitory subpopulations as described in the main text. The total number of neurons in the excitatory and inhibitory neurons was 6800 and 1200 respectively. There were 2040 *E*_1_, 2040 *E*_2_, 2720 *E*_non_, 360 *I*_1_, 360 *I*_2_ and 480 *I*_non_ neurons. The membrane potential $\left(V_{m}^{i}\right)$ of each neuron *i* evolved according to the following equation:

(4) $$\tau_{m}^{i}\frac{dV_{m}^{i}}{dt}=-\left(V_{m}^{i}-V_{\text{rest}}\right)+I_{\text{ext}}^{i}+I_{\text{syn}}^{i},$$

where $\tau_{m}^{i}$ is the membrane time constant (20 ms for excitatory neurons and 10 ms for inhibitory neurons), *V*_rest_ = −60 mV is a resting potential, $I_{\text{ext}}^{i}$ is the external input and $I_{\text{syn}}^{i}$ is the synaptic input from other neurons in the network. The synaptic current $I_{syn}^{i}$ satisfies

(5) $$\frac{I_{\text{syn}}^{i}}{\text{dt}}=\frac{-I_{\text{syn}}^{i}}{\tau_{\text{syn}}}+\sum_{j=1}^{N}J_{\text{ij}}\sum_{t_{j}}\delta \left(t-t_{j}\right)$$

where *J*_*ij*_ is the synaptic strength from neuron *j* to neuron *i*, *τ*_syn_ = 2 ms is the synaptic time constant, and *t*_*j*_’s are the spike times of neuron *j*. The momentary firing rate of neuron *i* was given by a sigmoidal transfer function of its membrane potential:

(6) $$r\left(V_{m}^{i}\right)=\frac{30}{1+\text{exp}\left[-\left(\left(V_{m}^{i}-V_{\text{rest}}\right)-15\right)/3\right]}(\text{Hz}).$$

At each time step, neuron *i* emitted a spike with probability $r\left(V_{m}^{i}\right)$, where *dt* = 0.1 ms is the time step size.

The synaptic strength *J*_*ij*_ equals *W*_*αβ*_ with probability *p*_*αβ*_, where *α, β* ∈{*E, I*} denote the cell types of neurons *i* and *j* respectively. There is no self-connection, i.e. *J*_*ii*_ = 0. The connection strengths and connection probabilities are shown in Table 1.

The external current, $I_{\text{ext}}^{i}$, has four components,

(7) $$I_{\text{ext}}^{i}=I_{\text{base}}+I_{\text{context}}^{i}+I_{\text{sound}}^{i}(t)+I_{\text{photostim}}^{i}(t),$$

where *I*_base_ = 5 mV/ms is a constant baseline input, $I_{\text{context}}^{i}$ (mV/ms) is a constant input that is only present in the active context and is zero in the passive context, $I_{\text{sound}}^{i}(t)$ (mV/ms) is the sound input that is turned on at time *t*_on_, and $I_{\text{photostim}}^{i}(t)$ (mV/ms) is the photostimulus input that has the same onset time as sound and lasts for 100 ms.

(8) $$I_{\text{sound}}^{i}(t)=\left\{\begin{array}{ll} A^{i}\;\text{exp}\left(-\left(t-t_{\text{on}}\right)/500\right), & t\geq t_{\text{on}}\\ 0, & t<t_{\text{on}} \end{array}.\right.$$

(9) $$I_{\text{photostim}}^{i}(t)=\left\{\begin{array}{ll} S^{i}, & t_{\text{on}}\leq t\leq t_{\text{on}}+100\\ 0, & \text{else} \end{array}.\right.$$

The context current ${(I}_{\text{context}}^{i})$ is applied to 2000 neurons randomly chosen from all populations in the active context. This choice is motivated by the findings in Extended Data Figure 8(i), which show that roughly 25 − 30% of sound-modulated neurons have positive engagement indices. The strength of the context current is sampled from a gamma distribution Γ(*α* = 5*,θ* = 1). For left sound, the strength of sound is *A*^*i*^ = 5 for *E*_1_ and *I*_1_ neurons, and *A*^*i*^ = 4.25 for *E*_2_ and *I*_2_ neurons. For right sound, the strength of sound is *A*^*i*^ = 4.25 for *E*_1_ and *I*_1_ neurons, and *A*^*i*^ = 5 for *E*_2_ and *I*_2_ neurons. There is no sound input to *E*_non_ and *I*_non_ neurons, i.e. *A*^*i*^ = 0 for *i* ∈{*E*_non_*, I*_non_}. The strength of photostimulation current (*S*^*i*^) is sampled from a gamma distribution, Γ(*α* = 7*,θ* = 1), and is applied to half of the *E*_*non*_ and half of the *I*_*non*_ populations, selected at random. We impose the constraint that no neuron receives both photostimulation and context inputs.

We studied two stimulus conditions (sound alone, sound+photostimulus) and two context conditions (active, passive). For each stimulus and context condition, there were 1000 trials of 1500 ms duration each. The onset of sound and photostimulation was *t*_on_ = 1000 ms. The sound modulation index was calculated using the trial-averaged firing rates during a 700 ms time window before sound onset (*R*_pre_) and rates during a 300 ms time window after sound onset (*R*_post_) in the sound alone condition, SMI = *R*_post_ − *R*_pre_. The photostimulus modulation index was calculated by comparing the rates during a 300 ms time window after stimulus onset in the sound+photostimulus condition and the rates during the same time window in the sound alone condition, PMI = *R*_sound+stim_ − *R*_sound_.

The model was simulated with forward Euler scheme with time step size 0.1 ms. All simulations were written in a combination of C and MATLAB (MATLAB R 2022b, MathWorks). All model analysis was conducted in Python.

## Extended Data

**Extended Data 1:**
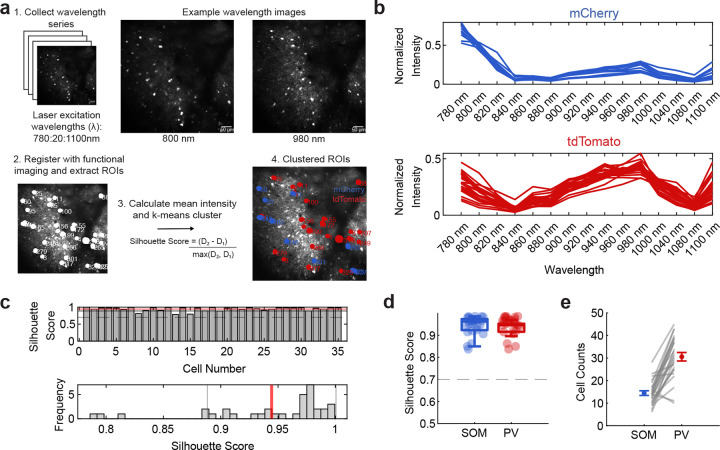
**, related to** Figure 1 Spectral clustering of red-labeled inhibitory neurons across datasets. (a) Workflow for spectral clustering of red-labeled neurons. (1) A wavelength excitation series (780–1100 nm) was acquired during the imaging session, with 800 nm and 980 nm providing maximal separation between mCherry and tdTomato signals. (2) Images were registered to the functional images to extract ROIs. (3) Mean pixel intensity for each ROI was calculated for each wavelength, and used (4) for *k*-means clustering. Silhouette scores quantified the separation between mCherry and tdTomato clusters (*D*_1_ = mean distance to own cluster; *D*_2_ = mean distance to nearest other cluster). (b) Normalized fluorescence intensity profiles across wavelengths (780–1100 nm) for individual cells after clustering in an example dataset. Each line represents one red-labeled neuron from the example dataset. *k*-means clustering was performed using a specified combination of wavelengths (780, 800, 820, 940, 960 nm) on these spectral profiles to classify cells as mCherry or tdTomato. (c) (top) Silhouette scores for all red-labeled cells in the example dataset, quantifying clustering confidence (red line indicates the mean, dotted line is the exclusion threshold). (bottom) Frequency of silhouette scores. Red line indicates the mean, black lines indicate the standard deviation. (d) Mean silhouette scores of SOM and PV neurons across datasets from clustering (0.9448 ± 0.0425 SOM, 0.9329 ± 0.0332 PV; mean ± s.e.m., *n* = 25 datasets from 6 mice). Dotted line at 0.7 indicates exclusion threshold. (e) Distribution of cell counts per cell type (SOM vs. PV) across datasets (14 ± 1 SOM, 31 ± 2 PV; mean ± s.e.m., *n* = 25 datasets from 6 mice). Gray lines link values from the same imaging session.

**Extended Data Figure 5:**
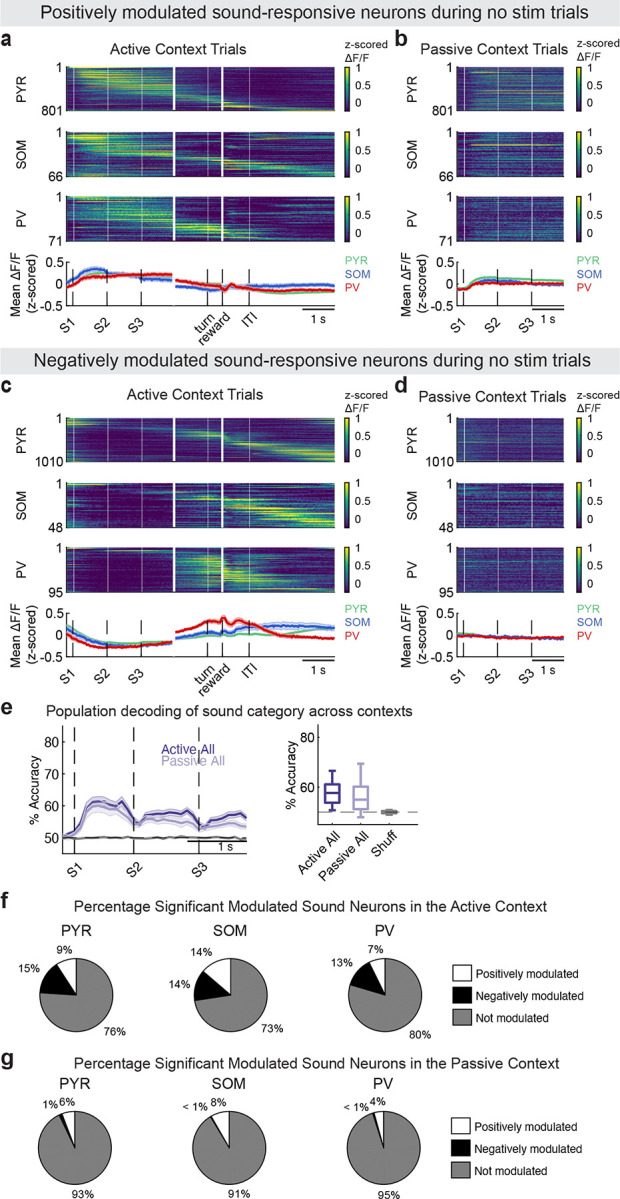
**, related to** Figure 2. Sound responses in active and passive listening. (a,b) Top: Trial-averaged, z-scored activity of significantly positively modulated sound-responsive neurons (PYR *n* = 801, SOM *n* = 66, PV *n* = 71), sorted by time of peak mean activity during the active condition, shown for (a) active and (b) passive control trials (trials without photostimulation, PYR *n* = 801, SOM *n* = 66, PV *n* = 71). Bottom: Trial-averaged activity of each cell type in active (a) and passive (b) (mean ± s.e.m. across neurons). (c,d) Top: Same as (a,b) but for significantly negatively modulated sound-responsive neurons (PYR *n* = 1010, SOM *n* = 48, PV *n* = 95), shown for (c) active and (d) passive control trials. Bottom: Trial-averaged activity of each cell type in active (c) and passive (d) contexts (mean ± s.e.m. across neurons). (e) *Left*: Classification accuracy of SVM decoders trained on all neurons in active and passive contexts. *Right*: Mean classification accuracy in the 450 ms after sound-event onset (*n* = 23 datasets). No significant differences across contexts (*p* = 0.4191). (f,g) Mean percentage of significantly modulated sound neurons by cell type in the active (f) and passive (g) contexts across datasets (*n* = 24). Exact mean % ± s.d. are provided in Extended Table 2. For all panels, n.s. means no significance; *, *p* < 0.05; **, *p* < 0.01; ***, *p* < 0.001; ****, *p* < 0.0001.

**Extended Data Figure 6:**
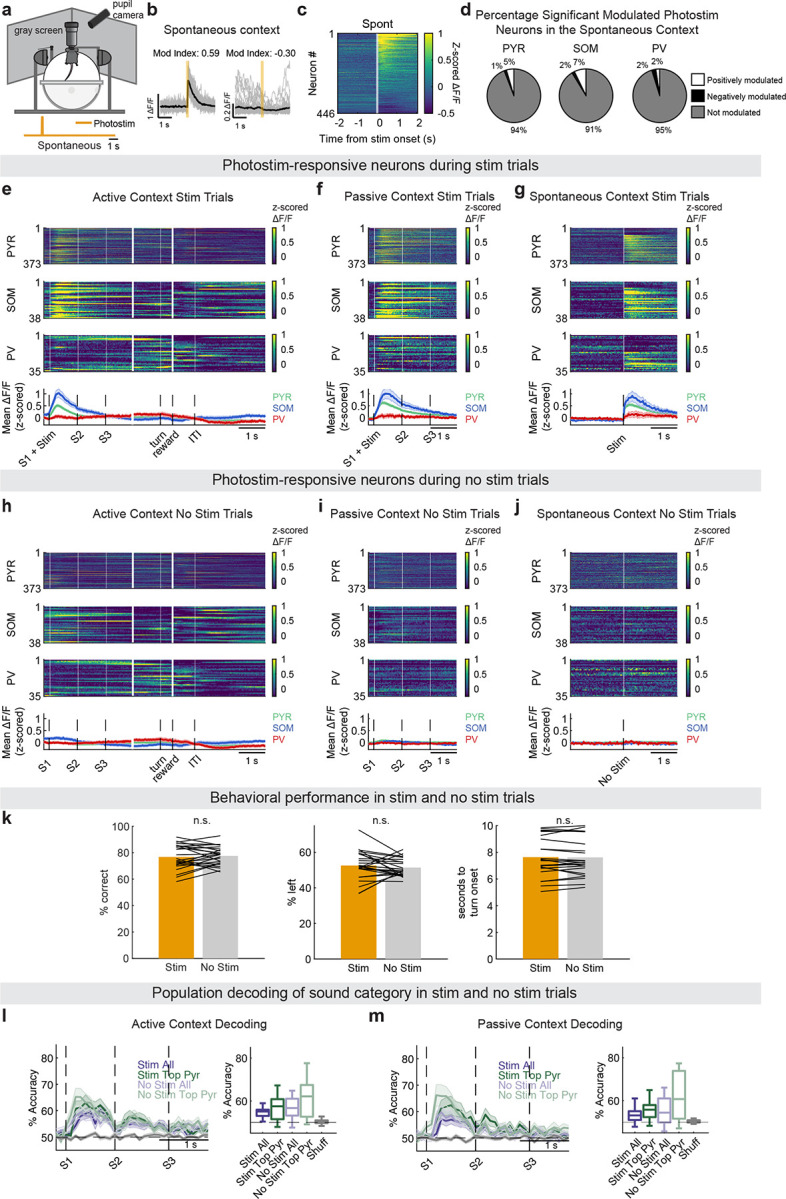
**, related to** Figure 2. Photostimulation responses in spontaneous, active listening, and passive listening contexts. (a) Spontaneous context. Imaging was performed in this context without presenting sound stimuli. Photostimulations were delivered at 10-second intervals. (b) Example positively and negatively modulated neurons during the spontaneous context (computed using the SMI). Black lines: mean across trials; gray lines: individual trials. (c) Trial averaged activity heatmap of photostimulus-responsive neurons, aligned to photostimulation onset, during the spontaneous context (*n* = 446 neurons). (d) Mean percentage of neurons significantly responsive to the photostimulus in the spontaneous context across datasets (*n* = 24). Exact mean % ± s.d. are provided in Extended Table 2. (e) *Top*: Trial-averaged, z-scored activity of all significant photostimulation-responsive neurons (PYR *n* = 373, SOM *n* = 38, PV *n* = 35 neurons), during photostimulus+sound trials in the active listening condition. Neurons were sorted by time of peak mean activity during the spontaneous context. *Bottom*: Trial-averaged activity across all neurons in each cell typein sound+photostim trials in the active listening context (mean ± s.e.m. across neurons).(f) As in (e), but activity of photostimulation-responsive neurons during photostimulus+sound trials in the passive listening context. (g) As in (e-f), but activity during trials in photostimulus trials in the spontaneous context. Neurons in (e-g) were sorted by time of peak activity in (g). (h) As in (e), but activity during no photostimulation control trials (0 power LED) in the active listening context. (i) As in (h), in the passive listening context. (j) As in (h), but in the spontaneous context. Neurons in h-j were sorted by time of peak activity in (g). (k) Behavioral performance metrics comparing photostimulation (orange) and no photostimulation (gray) trials during the active task. *Left*: Percent correct responses. *Middle*: Percent of leftward choices. *Right*: Reaction times (seconds to turn onset from the start of the trial). Black lines link values from the same imaging session (*n* = 24). No significant behavioral differences were observed across conditions (*p*_correct_ = 0.5677, *p*_left_ = 0.2652, *p*_sec to turn_ = 0.9317; sign-rank test). (l) Population decoding accuracy of sound category across time for active context, comparing photostimulation (dark) and no photostimulation (light) trials (mean ± s.e.m. across datasets). *Right*: Box plots summarize mean decoding accuracy across 450 ms of the first sound repeat (S1) across datasets. Shuffled control (gray) reflects chance level. No significant difference in decoding performance was observed between photostimulation and no photostimulation trials (*n* = 14 datasets, *p*_all_ = 0.92491*, p*_top pyr_ = 0.85101; paired permutation test). (m) Same as (l), but for passive context. No significant impact of photostimulation on decoding accuracy was observed (*n* = 14 datasets, *p*_all_ = 0.55864*, p*_top pyr_ = 0.1005; paired permutation test)

**Extended Data Figure 7:**
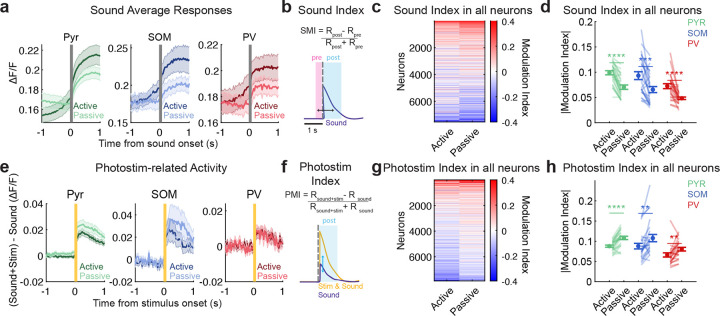
**, related to** Figure 2. Inclusion of all neurons yields similar results as when limiting to significantly responsive neurons. (a) Trial-averaged responses of positively modulated neurons in active and passive contexts (mean ± s.e.m. across datasets, Pyr *n* = 25, SOM *n* = 22, PV *n* = 23). (b) Definition of sound modulation index. (c) Heatmap of sound modulation indices of all recorded neurons. Neurons were sorted by their mean modulation index across contexts (*n* = 7885 neurons). (d) Mean absolute value sound modulation index including all recorded neurons in each cell type (paired permutation test: *p*_pyr_ < 0.0001, *p*_som_ = 0.0003, *p*_pv_ < 0.0001). Lines link values from the same imaging session (Pyr *n* = 25, SOM *n* = 25, PV *n* = 25). (e) Same as (a), but neurons with positive trial averaged difference in responses during sound+photostimulation and sound-only trials (mean ± s.e.m. across datasets, Pyr *n* = 24, SOM *n* = 24, PV *n* = 24). (f) Definition of photostim modulation index. (g) Heatmap of photostimulation modulation indices of all recorded neurons, sorted by mean modulation index across contexts (*n* = 7545 neurons). (h) Same as (d), but for photostimulation modulation index (paired permutation test: *p*_pyr_ < 0.0001, *p*_som_ = 0.0023, *p*_pv_ = 0.0027; mean ± s.e.m. across datasets, Pyr *n* = 24, SOM *n* = 24, PV *n* = 24). For all panels, n.s. means no significance; *, *p* < 0.05; **, *p* < 0.01; ***, *p* < 0.001; ****, *p* < 0.0001.

**Extended Data Figure 8:**
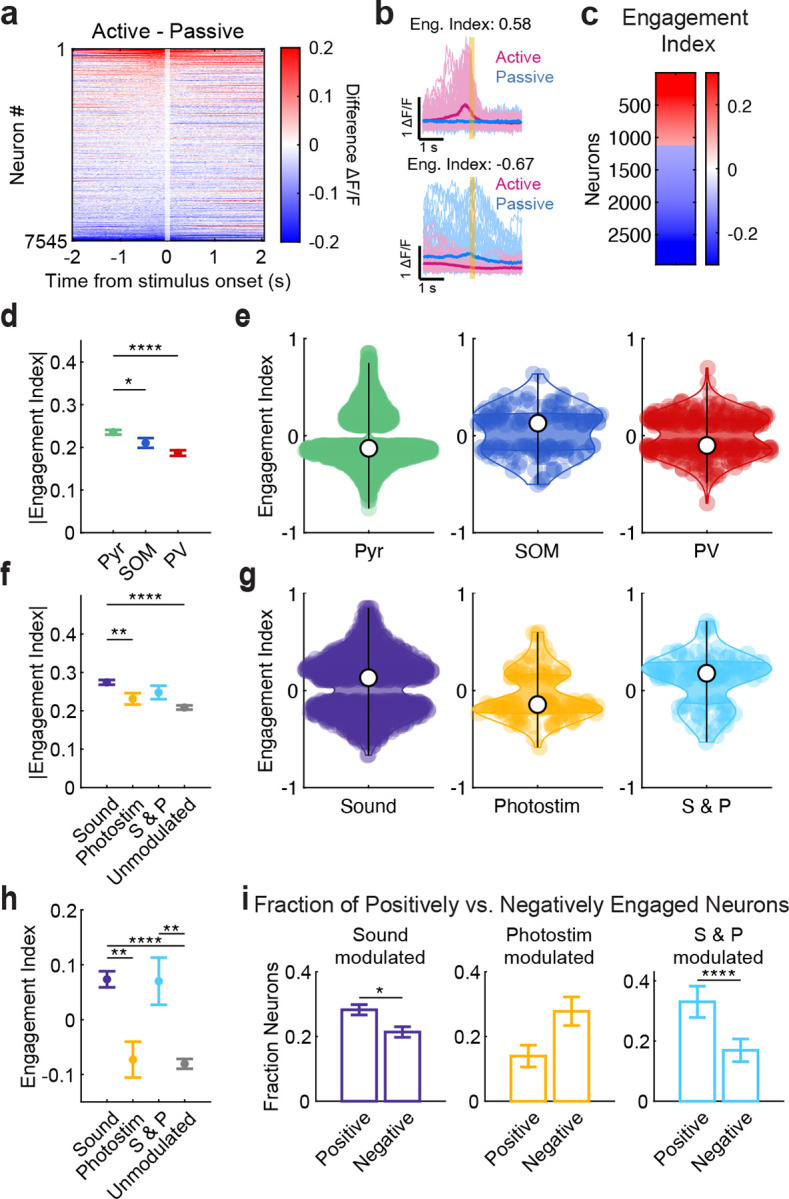
**, related to** Figure 3. Pre-stimulus activity differences across contexts, quantified with the Engagement Index. (a) Differences between active and passive trial-averaged activity, aligned to stimulus onset, sorted by magnitude of activity in the pre-stimulus window. Positive values (red) indicate higher baseline activity in active trials. (b) Example of a neuron with positively modulated pre-stimulus activity (top) and negatively modulated pre-stimulus activity (bottom). Bold lines show session means; thin traces are individual trials. The Engagement Modulation Index (Equation 3) was calculated by comparing mean pre-stimulus activity between active and passive conditions. (c) Heatmap of Engagement Index (EI) of all neurons with significant EI (*n* = 2963). (d) Mean absolute value of the Engagement Index in each cell type for significantly engaged neurons (paired permutation test: *p*_pyr–som_ = 0.0127, *p*_pyr–pv_ < 0.00001, *p*_som–pv_ = 0.0352; mean ± s.e.m. across datasets; Pyr *n* = 24, SOM *n* = 24, PV *n* = 24. Only datasets with at least 1 significant neuron were included). (e) Distributions of Engagement Index across neurons in each cell type, only for significantly engaged neurons. White dots mark medians; colored dots are individual neurons (PYR *n* = 2553, SOM *n* = 140, PV *n* = 270 neurons). (f) Mean absolute value of the Engagement Index in each functional group for significantly engaged neurons (paired permutation test: *p*_sound–photostim_ = 0.0016, *p*_sound–unmodulated_ < 0.0001; mean ± s.e.m. across datasets, sound *n* = 24, photostim *n* = 23, S&P *n* = 21, unmodulated *n* = 24. Only datasets with at least 1 significant neuron were included). (g) Distribution of Engagement Index in each functional group, only for significantly engaged neurons (Sound = 996, Photostim = 104, S&P = 100). (h) Mean raw Engagement Index in each functional group, only for significantly engaged neurons (paired permutation test: *p*_sound–photostim_ = 0.0020, *p*_sound–unmodulated_ < 0.0001, *p*_S&P–unmodulated_ = 0.0029; mean ± s.e.m. across datasets, sound *n* = 24, photostim *n* = 23, S&P *n* = 21, unmodulated *n* = 24. Only datasets with at least 1 significant neuron were included). (i) Fraction of neurons with significant positive or negative engagement index within each functional group (paired permutation test: *p*_sound_ = 0.01450 *n* = 24, *p*_photostim_ = 0.0515 *n* = 24, *p*_S&P_ =< 0.00001 *n* = 23; mean ± s.e.m. across datasets). For all panels, n.s. means no significance; *, *p* < 0.05; **, *p* < 0.01; ***, *p* < 0.001; ****, *p* < 0.0001. Exact values are provided in Extended Table 3.

**Extended Data Figure 9:**
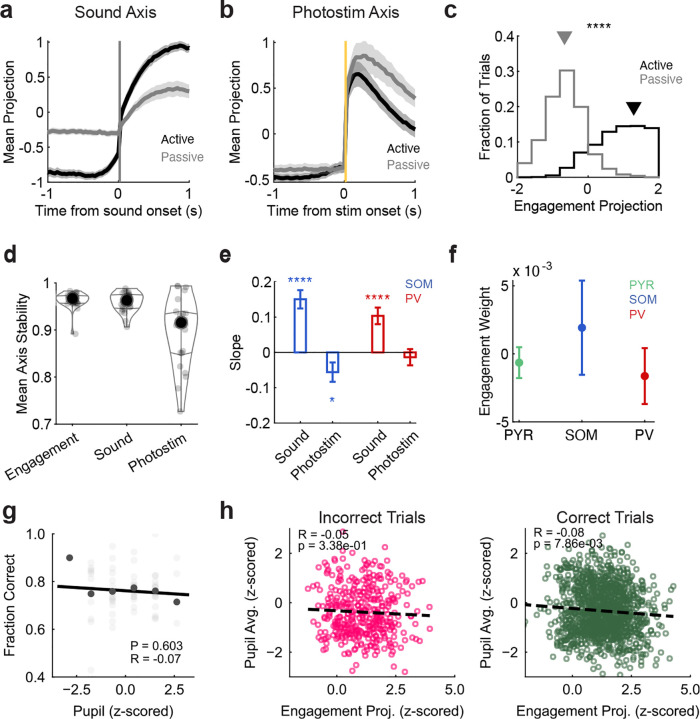
**, related to** Figure 3. Axis projections, cell type contributions, and pupil relationships. (a) Mean trial-aligned population activity projections onto the sound axis (mean ± s.e.m. across datasets, *n* = 24). (b) Mean trial-aligned population activity projections onto the photostimulation axis (mean ± s.e.m. across datasets, *n* = 24). (c) Distribution of engagement projections in active (black) vs. passive (gray) trials, showing a significant rightward shift in the active condition (*p* < 0.0001, permutation test). (d) Axis stability across folds per dataset (cosine similarity of axis weights). Black circles are sessions; gray dots are session means. (e) Slopes of linear regression relating engagement projection to sound or photostimulation projection (defined across all neurons) with engagement axes constructed using only SOM (blue) or PV (red) neurons (*p*_sound,SOM_ = 7.16–10^−9^, *p*_photostim,SOM_ = 0.0417, *p*_sound,PV_ = 1.14 × 10^−5^, *p*_photostim,PV_ = 0.5514). (f) Contribution of each cell type to the engagement axis (paired permutation test: *p*_pyr–som_ = 0.0039, *p*_pyr–pv_ = 0.0001, *p*_som–pv_ = 0.3747; mean ± s.e.m. across datasets, *n* = 24). (g) Behavioral performance (fraction correct) as a function of average pupil size in the last 300 ms of the ITI from the previous trial. Each dot is a session; bins ≥5 trials were used to compute session means. Black line is the linear regression across datasets; dark gray dots are session means. (h) Trial-level relationships between engagement projection and pupil averages. Each dot is a trial (*n* = 373 correct trials, *n* = 1251 correct trials from 14 datasets, 4 mice). Black line represents the fitted slope from a linear regression model. For all panels, n.s. means no significance; *, *p* < 0.05; **, *p* < 0.01; ***, *p* < 0.001; ****, *p* < 0.0001.

**Extended Data Figure 10:**
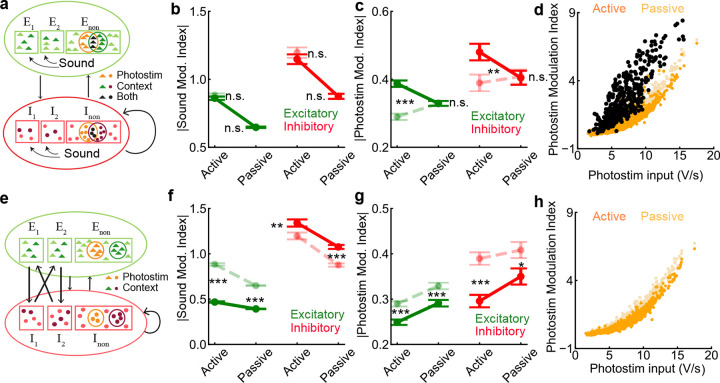
*Top row*: (a) Schematic of the network model where some neurons in the subpopulations *E*_non_ and *I*_non_ are allowed to receive both contextual and photostimulus inputs. The neurons that receive only photostimulation inputs are in orange, and neurons that receive only context inputs are in dark green (excitatory) or dark red (inhibitory). Neurons that receive both photostimulation and context inputs are in black. The network connectivity and other parameters are the same as in the main text. (b) Average sound modulation index magnitude for the excitatory (solid green) and inhibitory (solid red) populations in the active and passive contexts. For both excitatory and inhibitory populations, the absolute average modulation index with respect to sound was larger in the active context, consistent with the data (Figure 2g). The change of the average magnitude of sound modulation indices from the passive to active context is 0.21 for excitatory and 0.27 for inhibitory neurons. Dashed red and green lines are the average absolute values of the sound modulation index of the model used to generate Figure 4. There is no significant difference between the solid and dashed lines (*p* > 0.1 for both populations in both active and passive contexts). (c) Same as (b) for the photostimulus modulation index. For both excitatory and inhibitory populations, the average absolute modulation index with respect to photostimulation was larger in the active context, inconsistent with the data (Figure 2k). The change of the average magnitude of photostimulation modulation indices from the passive to active context is 0.058 for excitatory and 0.076 for inhibitory neurons. Note the contrast with the results from Figure 4 (dashed lines) (*p* > 0.1 for passive photostim for excitatory and inhibitory neurons. *p* = 2 × 10^−4^ for excitatory and 0.003 for inhibitory neurons in the active photostim case). (d) Scatter plot of the photostim modulation index in the active (darker orange) and passive (lighter orange) contexts for neurons that received photostimulation inputs. Neurons that received both photostimulation and context inputs had much larger photostim modulation indices in the active context (black dots), indicating that the contextual inputs enhanced the responses to photostimulation, in contrast to the model used in the main text. *Bottom row*: Comparison of a model with structured connectivity with the model in Figure 4. (e) Model schematic for a network with opponent inhibition motif [8]. Here, the *E*_1_ → *I*_1_ and *E*_2_ → *I*_2_ connections (black arrows) are stronger and denser compared to other E→I connections (grey arrows), and *I*_1_ → *E*_2_ and *I*_2_ → *E*_1_ connections (black arrows) are stronger and denser compared to other I→E connections (grey arrows). Thus, *E*_1_ preferentially activates *I*_1_ which preferentially inhibits *E*_2_, and *E*_2_ preferentially activates *I*_2_ which preferentially inhibits *E*_1_. The modified connection probabilities were $p_{E_{1}\to I_{1}}=p_{E_{2}\to I_{2}}=0.044$ and $p_{I_{1}\to E_{2}}=p_{I_{2}\to E_{1}}=0.26$, and the modified connection strengths were $W_{E_{1}\to I_{1}}=W_{E_{2}\to I_{2}}=11\;\text{mV}$ and $W_{I_{1}\to E_{2}}=W_{I_{2}\to E_{1}}=-3.9\;\text{mV}$. All other parameters were the same as the model in Figure 4. The neurons that received photostimulation were distinct from those that received context input, as in Figure 4. (f, g) Same as (b, c). The average magnitude of the sound modulation index was greater in the active context and the average magnitude of the photostimulation modulation index was lower in the active context, consistent with data (Figure 2g,k). The changes of the average magnitude of sound modulation indices from the passive to active context are 0.076 (excitatory) and 0.26 (inhibitory), and the changes of the average magnitude of photostimulation modulation indices from the passive to active context are −0.042 (excitatory) and −0.054 (inhibitory). (h) Photostim modulation indices of neurons that received photostimulation in the active (dark orange) and passive (light orange) contexts. The photostim modulation index in the active context was lower than that in the passive context across photostimulus input strengths. For all panels, n.s. means not significant, ⋆ corresponds to *p* < 0.05; ⋆⋆, *p* < 0.01; ⋆–⋆–⋆, *p* < 0.001; ⋆⋆⋆⋆, *p* < 0.0001.

## Extended Data

**Table:** Statistical summary for Figure 1.

Group	N	Mean	Standard Deviation	SEM	p value	Effect Size
Figure 1g						
Fraction of Pyr neurons with peaks during S1	25	0.098379128	0.030721946	0.006144389		
Fraction of SOM neurons with peaks during S1	25	0.134049567	0.132899515	0.026579903		
Fraction of PV neurons with peaks during S1	25	0.094000468	0.086519777	0.017303955		
Fraction of Pyr neurons with peaks during S2	25	0.056842771	0.015545807	0.003109161		
Fraction of SOM neurons with peaks during S2	25	0.027617952	0.052127888	0.010425578		
Fraction of PV neurons with peaks during S2	25	0.063168564	0.047093482	0.009418696		
Fraction of Pyr neurons with peaks during S3	25	0.108417242	0.043263191	0.008652638		
Fraction of SOM neurons with peaks during S3	25	0.07436298	0.098132764	0.019626553		
Fraction of PV neurons with peaks during S3	25	0.108193449	0.070933195	0.014186639		
Fraction of Pyr neurons with peaks during Turn	25	0.256929218	0.051582023	0.010316405		
Fraction of SOM neurons with peaks during Turn	25	0.162322732	0.117233691	0.023446738		
Fraction of PV neurons with peaks during Turn	25	0.277375463	0.143723537	0.028744707		
Fraction of Pyr neurons with peaks during Reward	25	0.206224616	0.059396654	0.011879331		
Fraction of SOM neurons with peaks during Reward	25	0.204602702	0.145097618	0.029019524		
Fraction of PV neurons with peaks during Reward	25	0.275194025	0.133927974	0.026785595		
Fraction of Pyr neurons with peaks during ITI	25	0.223401018	0.046791955	0.009358391		
Fraction of SOM neurons with peaks during ITI	25	0.379313559	0.189613704	0.037922741		
Fraction of PV neurons with peaks during ITI	25	0.160448796	0.110608185	0.022121637		
Paired permutation test						
S1 Pyr vs SOM					0.162583742	−0.035670439
S1 Pyr vs PV					0.777522248	0.00437866
S1 SOM vs PV					0.209679032	0.040049099
S2 Pyr vs SOM					0.01829817	0.02922482
S2 Pyr vs PV					0.495450455	−0.006325792
S2 SOM vs PV					0.00759924	−0.035550612
S3 Pyr vs SOM					0.072892711	0.034054262
S3 Pyr vs PV					0.98460154	0.000223793
S3 SOM vs PV					0.188881112	−0.033830469
Turn Pyr vs SOM					0.00039996	0.094606486
Turn Pyr vs PV					0.431356864	−0.020446244
Turn SOM vs PV					0.00109989	−0.115052731
Reward Pyr vs SOM					0.95660434	0.001621914
Reward Pyr vs PV					0.00559944	−0.068969409
Reward SOM vs PV					0.052394761	−0.070591323
ITI Pyr vs SOM					0.00029997	−0.155912541
ITI Pyr vs PV					0.01339866	0.062952222
ITI SOM vs PV					0.00019998	0.218864763
						
Figure 1i						
Pyr Sound Category Mean Accuracy (1)	23	57.62726449	5.306802483	1.10654481		
SOM Sound Category Mean Accuracy (2)	23	51.93579193	2.137574107	0.445715012		
PV Sound Category Mean Accuracy (3)	23	50.95613354	1.375163199	0.286741349		
Shuffled Sound Category Mean Accuracy (4)	23	49.99106366	0.401953804	0.083813162		
Kruskal Wallis (1–3)					1.81E-06	
Paired permutation test						
Pyr vs SOM					1.00E-04	5.691472567
SOM vs PV					1.00E-04	6.671130952
Pyr vs PV					0.074792521	0.979658385
Pyr vs Shuffled					1.64E-08	7.636200828
SOM vs Shuffled					0.000342023	1.944728261
PV vs Shuffled					0.021064015	0.965069876
						
Figure 1j						
Pyr Choice Mean Accuracy	23	72.51087215	6.89810994	1.438355352		
SOM Choice Mean Accuracy	23	55.79908644	3.523644654	0.7347307		
PV Choice Mean Accuracy	23	58.62314441	4.181557948	0.871915105		
Shuffled Choice Mean Accuracy	23	50.14100414	0.649516465	0.135433545		
Kruskal Wallis					2.62E-10	
Paired permutation test						
Pyr vs SOM					1.00E-04	16.71178571
SOM vs PV					1.00E-04	13.88772774
Pyr vs PV					0.0189981	−2.824057971
Pyr vs Shuffled					6.64E-09	22.36986801
SOM vs Shuffled					1.75E-08	5.658082298
PV vs Shuffled					7.56E-09	8.482140269
						
Figure 1k						
Pyr Outcome Mean Accuracy	19	68.85724624	7.610267457	1.745915093		
SOM Outcome Mean Accuracy	19	55.37326441	4.277003848	0.981211977		
PV Outcome Mean Accuracy	19	59.76044486	5.083060451	1.166134044		
Shuffled Outcome Mean Accuracy	19	50.13931078	0.482043857	0.110588445		
Kruskal Wallis					2.88E-07	
Paired permutation test						
Pyr vs SOM					1.00E-04	13.48398183
SOM vs PV					0.00019998	9.096801378
Pyr vs PV					0.00639936	−4.387180451
Pyr vs Shuffled					1.48E-07	18.71793546
SOM vs Shuffled					3.45E-06	5.233953634
PV vs Shuffled					1.48E-07	9.621134085
						
Extended Data 2						
Extended Data 2e						
Pyr subsampled Sound Category Mean Accuracy	23	51.8190528	1.464898875	0.305452531		
SOM subsampled Sound Category Mean Accuracy	23	51.87646222	2.090005365	0.435796244		
PV subsampled Sound Category Mean Accuracy	23	50.67878623	1.106845188	0.230793176		
All neurons Sound Category Mean Accuracy	23	57.66563147	4.850865314	1.011475339		
Top Pyr Sound Category Mean Accuracy	23	61.9842883	8.533968694	1.779455482		
Shuffled Sound Category Mean Accuracy	23	49.95906056	0.532980917	0.111134204		
Kruskal Wallis					8.40E-11	
Paired permutation test						
Pyr subsampled vs SOM subsampled (1 vs 2)					0.919008099	−0.05740942
SOM subsampled vs PV subsampled (2 vs 3)					0.01839816	1.197675983
PV subsampled vs All neurons (3 vs 4)					1.00E-04	−6.986845238
All neurons vs Top Pyr (4 vs 5)					0.03969603	−4.318656832
Pyr subsampled vs PV subsampled (1 vs 3)					0.0039996	1.140266563
SOM subsampled vs All neurons (2 vs 4)					1.00E-04	−5.789169255
PV subsampled vs Top Pyr (3 vs 5)					1.00E-04	−11.30550207
Pyr subsampled vs All neurons (1 vs 4)					1.00E-04	−5.846578675
SOM subsampled vs Top Pyr (2 vs 5)					1.00E-04	−10.10782609
Pyr subsampled vs Top Pyr (1 vs 5)					1.00E-04	−10.16523551
Pyr subsampled vs Shuffled					7.79E-06	1.859992236
SOM subsampled vs Shuffled					0.00022343	1.917401656
PV subsampled vs Shuffled					0.033088276	0.719725673
All neurons vs Shuffled					7.55E-09	7.706570911
Top Pyr vs Shuffled					9.95E-08	12.02522774
						
Extended Data 2f						
Pyr subsampled Choice Mean Accuracy	23	55.69876812	2.037571716	0.424863072		
SOM subsampled Choice Mean Accuracy	23	55.74687888	3.512682682	0.732444971		
PV subsampled Choice Mean Accuracy	23	55.64336957	2.60324557	0.542814225		
All neurons Choice Mean Accuracy	23	73.49774327	7.193057119	1.49985609		
Top Pyr Choice Mean Accuracy	23	72.20192547	9.813891357	2.046337806		
Shuffled Choice Mean Accuracy	23	49.97991977	0.667408276	0.139164246		
Kruskal Wallis					3.67E-15	
Paired permutation test						
Pyr subsampled vs SOM subsampled (1 vs 2)					0.95630437	−0.048110766
SOM subsampled vs PV subsampled (2 vs 3)					0.907209279	0.103509317
PV subsampled vs All neurons (3 vs 4)					1.00E-04	−17.85437371
All neurons vs Top Pyr (4 vs 5)					0.622037796	1.295817805
Pyr subsampled vs PV subsampled (1 vs 3)					0.938106189	0.055398551
SOM subsampled vs All neurons (2 vs 4)					1.00E-04	−17.75086439
PV subsampled vs Top Pyr (3 vs 5)					1.00E-04	−16.5585559
Pyr subsampled vs All neurons (1 vs 4)					1.00E-04	−17.79897516
SOM subsampled vs Top Pyr (2 vs 5)					1.00E-04	−16.45504658
Pyr subsampled vs Top Pyr (1 vs 5)					1.00E-04	−16.50315735
Pyr subsampled vs Shuffled					1.00E-04	5.718848344
SOM subsampled vs Shuffled					1.00E-04	5.76695911
PV subsampled vs Shuffled					1.00E-04	5.663449793
All neurons vs Shuffled					1.00E-04	23.5178235
Top Pyr vs Shuffled					1.00E-04	22.22200569
						
Extended Data 2g						
Pyr subsampled Outcome Mean Accuracy		55.68574561	2.834537956	0.650287605		
SOM subsampled Outcome Mean Accuracy		55.33131266	4.222179846	0.968634488		
PV subsampled Outcome Mean Accuracy		57.71281328	4.453811059	1.021774333		
All neurons Outcome Mean Accuracy		70.10236529	7.851788079	1.801323724		
Top Pyr Outcome Mean Accuracy		71.63782268	8.672268866	1.989554926		
Shuffled Outcome Mean Accuracy		49.80579574	0.590564526	0.135484794		
Kruskal Wallis					1.44E-12	
Paired permutation test						
Pyr subsampled vs SOM subsampled (1 vs 2)					0.766923308	0.354432957
SOM subsampled vs PV subsampled (2 vs 3)					0.102289771	−2.381500627
PV subsampled vs All neurons (3 vs 4)					1.00E-04	−12.38955201
All neurons vs Top Pyr (4 vs 5)					0.574842516	−1.535457393
Pyr subsampled vs PV subsampled (1 vs 3)					0.107989201	−2.027067669
SOM subsampled vs All neurons (2 vs 4)					1.00E-04	−14.77105263
PV subsampled vs Top Pyr (3 vs 5)					1.00E-04	−13.9250094
Pyr subsampled vs All neurons (1 vs 4)					1.00E-04	−14.41661967
SOM subsampled vs Top Pyr (2 vs 5)					1.00E-04	−16.30651003
Pyr subsampled vs Top Pyr (1 vs 5)					1.00E-04	−15.95207707
Pyr subsampled vs Shuffled					1.48E-07	5.879949875
SOM subsampled vs Shuffled					5.24E-06	5.525516917
PV subsampled vs Shuffled					1.48E-07	7.907017544
All neurons vs Shuffled					1.48E-07	20.29656955
Top Pyr vs Shuffled					1.48E-07	21.83202694
						
						
Extended Data 2i						
Pyr Sound Category % Informative	23	7.808803336	5.0854192	1.060383205		
SOM Sound Category % Informative	23	11.91181376	12.49368194	2.605112769		
PVSound Category % Informative	23	13.66217304	15.28462347	3.187064306		
Kruskal Wallis					0.633400719	
Pyr vs SOM					0.054194581	−4.103010426
Pyr vs PV					0.01579842	−5.8533697
SOM vs PV					0.497550245	−1.750359273
						
Pyr Sound Category Peak Information (bits)	474	0.079835186	0.030936766	0.001420973		
SOM Sound Category Peak Information (bits)	38	0.079634566	0.023546683	0.003819776		
PV Sound Category Peak Information (bits)	98	0.073792016	0.012472798	0.001259943		
Kruskal Wallis					0.598720803	
Pyr vs SOM					0.95010499	0.00020062
Pyr vs PV					0.03159684	0.006043169
SOM vs PV					0.077392261	0.005842549
						
Extended Data 2k						
Pyr Choice % Informative	23	25.34582595	10.53433805	2.196561325		
SOM Choice % Informative	23	40.48373068	30.12354327	6.281192975		
PV Choice % Informative	23	40.60731242	24.58841112	5.127038137		
Kruskal Wallis					0.147748177	
Pyr vs SOM					0.00279972	−15.13790473
Pyr vs PV					0.00019998	−15.26148647
SOM vs PV					0.96490351	−0.123581742
						
Pyr Choice Peak Information (bits)	1482	0.090138446	0.043751346	0.001136494		
SOM Choice Peak Information (bits)	124	0.082805889	0.022843986	0.002051451		
PV Choice Peak Information (bits)	290	0.081224347	0.02430018	0.001426956		
Kruskal Wallis					0.081567577	
Pyr vs SOM					0.04719528	0.007332557
Pyr vs PV					0.00019998	0.008914099
SOM vs PV					0.530546945	0.001581542
						
Extended Data 2m						
Pyr Outcome % Informative	19	29.47038014	13.75201654	3.154928968		
SOM Outcome % Informative	19	37.54018874	24.09528915	5.527838443		
PV Outcome % Informative	19	52.12940584	22.71177049	5.210437495		
Kruskal Wallis					0.006030831	
Pyr vs SOM					0.067193281	−8.069808599
Pyr vs PV					0.00019998	−22.6590257
SOM vs PV					0.00719928	−14.5892171
						
Pyr Outcome Peak Information (bits)	1423	0.087919844	0.047040764	0.001247016		
SOM Outcome Peak Information (bits)	114	0.079885382	0.019337358	0.00181111		
PV Outcome Peak Information (bits)	306	0.082441598	0.0248819	0.001422404		
Kruskal Wallis					0.844116088	
Pyr vs SOM					0.04079592	0.008034462
Pyr vs PV					0.03239676	0.005478245
SOM vs PV					0.333766623	−0.002556216

**Table:** Statistical summary for Figure 2.

Group	N	Mean	Standard Deviation	SEM	p value	Effect Size
Figure 2d						
Pyr sound evoked response (Active)	25	0.254482523	0.084379467	0.016875893		
Pyr sound evoked response (Passive)	25	0.202291932	0.030969723	0.006193945		
SOM sound evoked response (Active)	16	0.23985791	0.086492264	0.021623066		
SOM sound evoked response (Passive)	16	0.185602035	0.023668283	0.005917071		
PV sound evoked response (Active)	15	0.204998651	0.043274466	0.011173419		
PV sound evoked response (Passive)	15	0.170380217	0.014681899	0.00379085		
						
Figure 2g						
Pyr Absolute Sound Modulation Index (Active)	25	0.19752005	0.025503209	0.005100642		
Pyr Absolute Sound Modulation Index (Passive)	25	0.111744511	0.041557431	0.008311486		
SOM Absolute Sound Modulation Index (Active)	23	0.163258104	0.051611155	0.01076167		
SOM Absolute Sound Modulation Index (Passive)	23	0.089453747	0.04656811	0.009710122		
PV Absolute Sound Modulation Index (Active)	24	0.148825248	0.033082208	0.006752877		
PV Absolute Sound Modulation Index (Passive)	24	0.07940406	0.042466319	0.008668401		
						
Paired permutation test						
Pyr active vs Pyr passive					1.00E-04	0.085775539
SOM active vs SOM passive					1.00E-04	0.073804356
PV active vs PV passive					1.00E-04	0.069421188
						
Figure 2h						
Pyr photostimulation evoked response (Active)	24	0.099395879	0.066051669	0.01348274		
Pyr photostimulation evoked response (Passive)	24	0.155456686	0.108907274	0.022230604		
SOM photostimulation evoked response (Active)	11	0.112346211	0.070355786	0.021213068		
SOM photostimulation evoked response (Passive)	11	0.147464684	0.115719932	0.034890872		
PV photostimulation evoked response (Active)	12	0.027649106	0.027259851	0.007869241		
PV photostimulation evoked response (Passive)	12	0.044465738	0.053669458	0.015493038		
						
Figure 2k						
Pyr Absolute Photostim Modulation Index (Active)	24	0.182241938	0.048176152	0.009833916		
Pyr Absolute Photostim Modulation Index (Passive)	24	0.233085924	0.062275999	0.012712035		
SOM Absolute Photostim Modulation Index (Active)	14	0.167966611	0.107887495	0.028834146		
SOM Absolute Photostim Modulation Index (Passive)	14	0.227088293	0.033776523	0.126380175		
PV Absolute Photostim Modulation Index (Active)	16	0.108100718	0.016191339	0.064765355		
PV Absolute Photostim Modulation Index (Passive)	16	0.159863052	0.074623336	0.018655834		
						
Paired permutation test						
Pyr active vs Pyr passive					0.00109989	−0.050843985
SOM active vs SOM passive					0.00809919	−0.059121681
PV active vs PV passive					0.00589941	−0.051762334
						
Figure 2o						
Sound Modulated Correlations (Sound vs ΔStim) (Active)	24	−0.348943766	0.441835904	0.090189376		
Sound Modulated Correlations (Sound vs ΔStim) (Passive)	24	−0.345094568	0.305633425	0.062387162		
Photostim Modulated Correlations (Sound vs ΔStim) (Active)	23	0.339408389	0.440440133	0.091838116		
Photostim Modulated Correlations (Sound vs ΔStim) (Passive)	23	0.18500815	0.489760839	0.099972013		
S&P Modulated Correlations (Sound vs ΔStim) (Active)	19	0.066554809	0.684049672	0.156931758		
S&P Modulated Correlations (Sound vs ΔStim) (Passive)	19	0.152282366	0.595831826	0.144510444		
						
Unpaired permutation test vs zero						
Sound Modulated Correlations vs zero (Active)					0.0018	−0.348943766
Sound Modulated Correlations vs zero (Passive)					1.00E-04	−0.345094568
Sound Modulated Correlations vs zero (Active)					0.0015	0.339408389
Sound Modulated Correlations vs zero (Passive)					0.0799	0.18500815
Sound Modulated Correlations vs zero (Active)					0.6703	0.066554809
Sound Modulated Correlations vs zero (Passive)					0.2995	0.152282366
						
Extended Data 5						
Extended Data 5f						
Percentage positively modulated PYR neurons (Active)	25	9.1689375	3.0647156			
Percentage negatively modulated PYR neurons (Active)	25	14.8162609	6.358463			
Percentage unmodulated PYR neurons (Active)	25	76.0148016	8.3257665			
Percentage positively modulated SOM neurons (Active)	25	13.7248512	13.7438907			
Percentage negatively modulated SOM neurons (Active)	25	13.6904288	11.8528156			
Percentage unmodulated SOM neurons (Active)	25	72.58472	20.1730098			
Percentage positively modulated PV neurons (Active)	25	7.2664045	6.6328025			
Percentage negatively modulated PV neurons (Active)	25	13.1458067	10.6237647			
Percentage unmodulated PV neurons (Active)	25	79.5877888	14.7405436			
Extended Data 5g						
Percentage positively modulated PYR neurons (Passive)	25	5.7895125	4.7263657			
Percentage negatively modulated PYR neurons (Passive)	25	1.3195104	1.3443444			
Percentage unmodulated PYR neurons (Passive)	25	92.890977	5.6725214			
Percentage positively modulated SOM neurons (Passive)	25	8.2806263	10.0529864			
Percentage negatively modulated SOM neurons (Passive)	25	0.3636364	1.8181818			
Percentage unmodulated SOM neurons (Passive)	25	91.3557374	10.5768248			
Percentage positively modulated PV neurons (Passive)	25	4.2826338	5.586804			
Percentage negatively modulated PV neurons (Passive)	25	0.6403736	1.5472907			
Percentage unmodulated PV neurons (Passive)	25	95.0769925	6.2592387			
						
Extended Data 6						
Extended Data 6d						
Percentage positively modulated PYR neurons during spont	24	4.5518449	2.0500092			
Percentage negatively modulated PYR neurons during spont	24	1.362769	2.2029958			
Percentage unmodulated PYR neurons during spont	24	94.0853861	3.1439138			
Percentage positively modulated SOM neurons during spont	24	7.1775971	11.1261893			
Percentage negatively modulated SOM neurons during spont	24	1.5775156	3.2038642			
Percentage unmodulated SOM neurons during spont	24	91.2448873	11.215043			
Percentage positively modulated PV neurons during spont	24	2.3826136	3.0901106			
Percentage negatively modulated PV neurons during spont	24	2.4256236	3.9145629			
Percentage unmodulated PV neurons during spont	24	95.1917628	4.6676282			
						
Extended Data 7						
Extended Data 7a						
Pyr sound evoked response (Active)	25	0.208885526	0.048901616	0.009780323		
Pyr sound evoked response (Passive)	25	0.192614766	0.028201658	0.005640332		
SOM sound evoked response (Active)	22	0.230189725	0.075208209	0.016034444		
SOM sound evoked response (Passive)	22	0.197130152	0.043498418	0.009273894		
PV sound evoked response (Active)	23	0.201545088	0.044488351	0.009276462		
PV sound evoked response (Passive)	23	0.187000084	0.030061635	0.006268284		
						
Extended Data 7d						
Pyr Absolute Sound Modulation Index (Active)	25	0.09894836	0.020215527	0.004043105		
Pyr Absolute Sound Modulation Index (Passive)	25	0.070396995	0.021775337	0.004355067		
SOM Absolute Sound Modulation Index (Active)	25	0.093200559	0.038770192	0.007754038		
SOM Absolute Sound Modulation Index (Passive)	25	0.06557963	0.029878873	0.005975775		
PV Absolute Sound Modulation Index (Active)	25	0.072323427	0.024470054	0.004894011		
PV Absolute Sound Modulation Index (Passive)	25	0.048440321	0.012215142	0.002443028		
						
Paired permutation test						
Pyr active vs Pyr passive					1.00E-04	0.028551365
SOM active vs SOM passive					0.00029997	0.027620929
PV active vs PV passive					1.00E-04	0.023883106
						
Extended Data 7e						
Pyr photostimulation evoked response (Active)	24	0.013661473	0.009165338	0.001870867		
Pyr photostimulation evoked response (Passive)	24	0.020062993	0.01462161	0.002984624		
SOM photostimulation evoked response (Active)	24	0.017210756	0.031454446	0.006420612		
SOM photostimulation evoked response (Passive)	24	0.027970186	0.052855059	0.010788994		
PV photostimulation evoked response (Active)	24	0.0053699	0.008459944	0.001726879		
PV photostimulation evoked response (Passive)	24	0.004595398	0.012035955	0.002456829		
						
Extended Data 7h						
Pyr Absolute Photostim Modulation Index (Active)	24	0.182241938	0.048176152	0.009833916		
Pyr Absolute Photostim Modulation Index (Passive)	24	0.233085924	0.062275999	0.012712035		
SOM Absolute Photostim Modulation Index (Active)	14	0.167966611	0.107887495	0.028834146		
SOM Absolute Photostim Modulation Index (Passive)	14	0.227088293	0.033776523	0.126380175		
PV Absolute Photostim Modulation Index (Active)	16	0.108100718	0.016191339	0.064765355		
PV Absolute Photostim Modulation Index (Passive)	16	0.159863052	0.074623336	0.018655834		
						
Paired permutation test						
Pyr active vs Pyr passive				1.00E-04	−0.020459006	
SOM active vs SOM passive				0.00229977	−0.019865337	
PV active vs PV passive				0.00269973	−0.014235643	

**Table:** Statistical summary for Figure 3.

Group	N	Mean	Standard Deviation	SEM	p value	Effect Size
Figure 3b						
Pre Mean Sound Active	24	0.209116883	0.046590183	0.009510181		
Pre Mean Sound Passive	24	0.178918955	0.028435813	0.005804436		
Pre Mean Photostim Active	24	0.177522036	0.027971296	0.005709617		
Pre Mean Photostim Passive	24	0.192763699	0.050717967	0.010352762		
Pre Mean Sound&Photostim Active	23	0.189912181	0.07058161	0.014717283		
Pre Mean Sound&Photostim Passive	23	0.177442933	0.051726331	0.010785686		
Pre Mean Unmodulated Active	24	0.169596793	0.024215356	0.004942939		
Pre Mean Unmodulated Passive	24	0.180261102	0.023402612	0.004777038		
Paired permutation test						
Sound active vs Sound passive					1.00E-04	0.030197928
Photostim active vs Photostim passive					0.097990201	−0.015241662
Sound&Photostim active vs Sound&Photostim passive					0.264473553	0.012469248
Unmodulated active vs Unmodulated passive					1.00E-04	−0.010664309
						
Figure 3e						
Pyr absolute engagement axis weights	24	0.03013912	0.003921185	0.000800409		
SOM absolute engagement axis weights	24	0.023616375	0.008723032	0.001780581		
PV absolute engagement axis weights	24	0.021601068	0.00662802	0.001352939		
Kruskal Wallis					1.98E-05	
Paired permutation test						
Pyr vs SOM					0.00559944	0.006522745
Pyr vs PV					1.00E-04	0.008538052
SOM vs PV					0.382061794	0.002015307
						
Figure 3h						
Fraction Correct Bin 1 (−1 to −0.5)	4	0.601068376	0.179491758	0.089745879		
Fraction Correct Bin 2 (−0.5 to 0)	21	0.711855807	0.156128173	0.03406996		
Fraction Correct Bin 3 (0 to 0.5)	24	0.727538311	0.12779038	0.026085102		
Fraction Correct Bin 4 (0.5 to 1)	24	0.759571242	0.102673423	0.020958125		
Fraction Correct Bin 5 (1 to 1.5)	24	0.786759567	0.138117719	0.028193161		
Fraction Correct Bin 6 (1.5 to 2)	24	0.7883237	0.12399231	0.025309824		
						
Extended Data 8						
Extended Data 8d						
Pyr Absolute Engagement Index	24	0.235631709	0.028200123	0.005756326		
SOM Absolute Engagement Index	24	0.211261291	0.058020228	0.011843329		
PV Absolute Engagement Index	24	0.18625569	0.032292475	0.006591674		
Kruskal Wallis					8.14E-05	
Paired permutation test						
Pyr vs SOM					0.01269873	0.544913237
Pyr vs PV					1.00E-04	1.499723207
SOM vs PV					0.03519648	0.435013369
						
Extended Data 8e						
Pyr Engagement Index	2553	−0.035744985	0.270366069			
SOM Engagement Index	140	0.052452605	0.241081254			
PV Engagement Index	270	0.002647092	0.212140494			
						
Extended Data 8f						
Sound Modulated Absolute Engagement Index	24	0.274155611	0.030200997	0.006164753		
Photostim Modulated Absolute Engagement Index	23	0.218956441	0.07018298	0.014634163		
Sound & Photostim (S & P) Modulated Absolute Engagement Index	21	0.254507446	0.086261257	0.018823749		
Unodulated Absolute Engagement Index	24	0.208642856	0.027186294	0.005549379		
Kruskal Wallis					3.79E-05	
Paired permutation test						
Sound vs Photostim					0.00159984	0.810133552
Sound vs S & P					0.363763624	0.199053144
Sound vs Unmodulated					1.00E-04	1.840568203
Photostim vs S & P					0.373362664	−0.205008397
Photostim vs Unmodulated					0.389461054	0.182776859
S & P vs Unmodulated					0.03759624	0.488797228
						
Extended Data 8g						
Sound Modulated Engagement Index	996	0.062342852	0.303719835			
Photostim Modulated Engagement Index	104	−0.057496633	0.253078296			
Sound & Photostim (S & P) Modulated Engagement Index	100	0.118635734	0.279835159			
						
Extended Data 8h						
Sound Modulated Engagement Index	24	0.073493743	0.071804999	0.014657134		
Photostim Modulated Engagement Index	23	−0.072896203	0.157149191	0.032767872		
Sound & Photostim (S & P) Modulated Engagement Index	21	0.070089783	0.19726596	0.043046962		
Unmodulated Engagement Index	24	−0.080629398	0.043511817	0.008881812		
Kruskal Wallis					1.42E-07	
Paired permutation test						
Sound vs Photostim					0.0019998	0.85192978
Sound vs S & P					0.839216078	−0.044873977
Sound vs Unmodulated					1.00E-04	2.289940247
Photostim vs S & P					0.02389761	−0.554081141
Photostim vs Unmodulated					0.820517948	0.048293994
S & P vs Unmodulated					0.00289971	0.770556892
						
Extended Data 8i						
Percent Positively Engaged Sound Modulated	24	28.2691	7.8331	1.5989		
Percent Negatively Engaged Sound Modulated	24	21.3858	8.0201	1.6371		
Percent Positively Engaged Photostim Modulated	24	13.9435	16.5078	3.3696		
Percent Negatively Engaged Photostim Modulated	24	27.8081	21.6365	4.4165		
Percent Positively Engaged S & P Modulated	23	33.0378	25.4974	5.3166		
Percent Negatively Engaged S & P Modulated	23	16.908	18.513	3.8602		
						
Paired permutation test						
Positively Engaged Sound Modulated vs Negatively Engaged Sound Modulated					0.01449855	6.883287829
Positively Engaged Photostim Modulated vs Negatively Engaged Photostim Modulated					0.051494851	−13.86457293
Positively Engaged S & P Modulated vs Negatively Engaged S & P Modulated					1.00E-04	16.1298

## Figures and Tables

**Table 1:** Synaptic connection strengths and probabilities in the model.

Connection strength, *W*_*αβ*_				Connection probability, *p*_*αβ*_			
		from (*β*)				from (*β*)	
		E	I			E	I
to (*α*)	E	0	−2.6042	to (*α*)	E	0	0.1739
	I	5.5147	−7.8125		I	0.029	0.1333
